# Apoplastic proteomic reveals *Colletotrichum fructicola* effector CfXyn11A recognized by tobacco and suppressed by pear in the apoplast

**DOI:** 10.1186/s43897-025-00161-3

**Published:** 2025-07-07

**Authors:** Chenyang Han, Shutian Tao, Zhihua Xie, Fengquan Liu, Shaoling Zhang

**Affiliations:** 1 State Key Laboratory of Crop Genetics & Germplasm Enhancement and Utilization, Sanya Institute of Nanjing Agricultural University, Nanjing Agricultural University, Nanjing, 210095 China; 2 Institute of Plant Protection, Jiangsu Academy of Agricultural Sciences, Jiangsu Key Laboratory for Food Quality and Safety, State Key Laboratory Cultivation Base of Ministry of Science and Technology, Nanjing, 210014 China; 3 Department of Plant Pathology, Key Laboratory of Agricultural Microbiology, College of Agriculture, Guizhou University, Guiyang, 550025 China

**Keywords:** *Colletotrichum fructicola*, Pear, Apoplastic plant‒microbe interaction, Fungal effector, CfXyn11A

## Abstract

*Colletotrichum fructicola* is a hemibiotrophic fungal plant pathogen that transitions from biotrophic growth on living host tissue to necrotrophic tissue destruction. During the hemibiotrophic phase, numerous proteins are secreted into the apoplast, mediating host‒pathogen interactions. In this study, we employed apoplastic proteomics and RNA-seq to analyse the proteins secreted during the interaction between *C. fructicola* and pear. A secreted xylanase, CfXyn11A, was identified as a dual-function effector. In the nonhost *Nicotiana benthamiana*, it triggered immune responses, including reactive oxygen species production and programmed cell death. However, CfXyn11A evades detection in the host pear, enabling its role in cell wall degradation and nutrient acquisition. Genetic and biochemical assays confirmed that the immune-triggering function of CfXyn11A relies on its apoplastic localization and is independent of enzymatic activity. Additionally, we identified an aspartic protease-like protein, PbXIP1, in the pear apoplast, which binds CfXyn11A to suppress its enzymatic activity and virulence. This study highlights the role of apoplastic proteomics in elucidating the molecular mechanisms underlying plant immunity and pathogen virulence and emphasizes the contrasting outcomes of CfXyn11A in different host contexts. The findings provide new insights into the interplay between extracellular effectors and plant defense proteins during fungal infection.

**Supplementary Information:**

The online version contains supplementary material available at 10.1186/s43897-025-00161-3.

## Core

Apoplastic proteomics characterized the apoplastic proteins and their expression levels in the *C. fructicola*-pear interaction. CfXyn11A was recognized by tobacco but evaded recognition by pear. CfXyn11A is induced by plant xylan and participates in plant cell wall hydrolysis. Pear secretes PbXIP1 to inhibit CfXyn11A activity extracellularly and enhance disease resistance.

## Gene and accession numbers

BAX accession: NC_000019.10, INF1 accession: AY830094.1, CfXyn11A accession: NW_027094051.1, CfXyn10A accession: NW_027094055.1, NbWRKY7 accession AB445391.1, NbPTI5 accession: XM_019390590.1, NbCYP71D20 accession: KM410159.2, PbWRKY7 accession: XM_048575365.1, PbPTI5 accession: XM_048590971.1, PbCYP71D20 accession: XM_009380225.3, PbXIP1 accession: XM_009358484.3.

## Introduction

The cellular structure of plant leaves comprises a protective epidermis covering mesophyll and vasculature tissues (Ye 2002). Beyond the solid mass of cells, the leaf interior contains extracellular spaces (Sattelmacher, 2001). The apoplast encompasses the interfibrillar and intermicellar spaces of cell walls, the xylem, and the gas- and water-filled intercellular spaces (Sattelmacher, 2001; Aung et al. 2018). This plant apoplast serves as a critical environment for signal exchange, nutrient uptake, and microbial adaptation and infection (Dora et al. 2022). In plant‒microbe interaction studies, the apoplast’s definition varies based on pathogen lifestyles (Wang et al. 2020). In plant‒fungal interaction systems, the apoplast typically includes intercellular spaces, plant cell walls, and the host‒pathogen interfaces formed between the plant cytoplasm and specialized fungal infection structures (Kankanala et al. 2007; Kwaaitaal et al. 2017). During infection by fungi and oomycetes, the apoplast serves as the initial interaction site, where pathogens reside within the host, particularly for certain hemibiotrophic pathogens (Giraldo and Valent 2013; Dora et al. 2022). Thus, the apoplast represents an active zone of close communication between plants and pathogen.

The apoplastic interactions between plants and microbes encompass several crucial processes, including pathogen attacks on plant cell walls, activation of plant cell surface receptors in response to pathogen-/microbe-associated molecular patterns (PAMPs/MAMPs) or damage-associated molecular patterns (DAMPs), suppression of plant immunity by pathogen effectors, and plant-secreted proteins counteracting pathogen effectors, among others (Macho and Zipfel 2014; Kubicek et al. 2014; Tariqjaveed et al. 2021; Wang et al. 2022b). While apoplastic effectors are crucial for plant‒pathogen interactions, the molecular mechanisms underlying key processes in extracellular immunity remain largely unelucidated.

Numerous pathogen apoplastic effectors are involved in pathogen virulence, with cell wall-degrading enzymes (CWDEs) being the most prominent (Kubicek et al. 2014). Pathogens secrete various CWDEs to break down the main structural polysaccharides of plant cell walls, including cellulose, hemicellulose, and pectin, to obtain nutrients and create space for growth (Cosgrove 2005). These CWDEs include cellulases, hemicellulases, pectinases, and other auxiliary enzymes (Kubicek et al. 2014). For instance, pathogens produce different types of cellulases to hydrolyze cellulose in the cell wall, which is then broken down by β-glucosidase into usable glucose from soluble cellodextrin oligomers (Teeri et al. 1997; Bhardwaj et al. 2021). These enzymes have been identified and studied in various pathogens and engineered strains, such as GH6 and GH7 cellulases from *Magnaporthe oryzae* (van Vu et al. 2012), GH5 endoglucanases from *Bacillus agaradhaerens* (Ma et al. 2020), and cellobiohydrolases from *Sclerotinia sclerotiorum* (SsdchA) (Chen et al. 2024). Besides cellulose, hemicellulose and pectin are significant targets. Xyloglucan-specific endoglucanase PsXEG1, a well-known hemicellulase, contributes to the full virulence of *Phytophthora sojae* (Ma et al. 2015, 2017). Silencing or deleting CWDEs that target pectin, such as the pectin methylesterase gene (*Bcpme1*) in *Botrytis cinerea*, the polygalacturonase gene (*Pcipg2*) in *Phytophthora capsici*, and the pectate lyase gene (*PlPeL1*) in *Peronophythora litchii*, significantly reduces microbial pathogenicity (Valette-Collet et al. 2003; Sun et al. 2009; Li et al. 2024).

At the plant cell surface, the perception of PAMPs/MAMPs by cell-surface pattern recognition receptors (PRRs) enables plants to rapidly respond to microbial challenges by pattern-triggered immunity (Boller and Felix 2009; Wan et al. 2019; Rocafort et al. 2020). Certain PAMPs, including flagellin, elongation factor Tu, and specific proteins secreted by pathogens, are recognized by PRRs (Kunze et al. 2004; Bigeard et al. 2015). Moreover, the induction of immune responses such as reactive oxygen species (ROS) production, immune marker gene expression, and callose deposition, which lead to programmed cell death (PCD) in plants, is a common feature of most proteinaceous MAMPs (Xu et al. 2021; Wang et al. 2022a). In addition to PRR-mediated plant immunity, plants secrete various proteins into the apoplast as a defensive measure (Wang et al. 2020). A significant proportion of the pathogenesis-related (PR) proteins identified to date have been categorized as plant apoplastic proteins (Sels et al. 2008). Among PRRs, PR protein chitinases (such as PR-3, 8, and 11) have been extensively researched; these hydrolases degrade chitin polymers by cleaving the β-1,4-glycosidic bonds of chitin molecules using two conserved glutamate residues (Gomez et al. 2002; Hong et al. 2002). Beyond directly attacking pathogenic tissues, some plant proteins, including proteases and hydrolase inhibitors, also interact directly with pathogen proteins in the apoplast (Juge et al. 2006; Jashni et al. 2015; Wang et al. 2020). For instance, among the 77 aspartic proteases in *Arabidopsis thaliana*, 51 are predicted to be located extracellularly in TAIR10 (Wang et al. 2019), and AtCDR1 overexpression can significantly enhance resistance to bacteria and fungi in both rice and *A. thaliana* (Prasad et al. 2009). To counter PsXEG1-mediated pathogenicity, soybean secretes the glucanase inhibitor protein (GIP) GmGIP1 into the apoplast, which directly interacts with PsXEG1 to reduce hexose production (Ma et al. 2017). A recent study demonstrated that the polygalacturonase-inhibiting protein from *Phaseolus vulgaris* interacts with the polygalacturonase from *Fusarium phyllophilum*, modifying the fungus’s ability to bind to cell wall polysaccharides and influencing substrate preferences and the types of degradation products generated, thereby enhancing immunity (Xiao et al. 2024). Therefore, constructing apoplastic protein repertoires in plant‒microbe communication is essential for studying the arms race between plants and pathogens in the apoplast.

Pear is one of the important economic fruit trees in the Rosaceae family (Liu et al. 2024). The interaction between pear and the fungal pathogen *Colletotrichum fructicola* offers an ideal model for investigating protein interactions in the plant apoplast, particularly during pathogen infection and immune response. *C. fructicola*, a hemibiotrophic pathogen, secretes various effectors into the apoplast during infection, making this system ideal for examining how plant immunity is mediated by extracellular proteins. The plant apoplast serves as a dynamic battleground of plant defense mechanisms and pathogen virulence strategies. Although pears have been less studied than model plants, their distinct immune landscape enables the exploration of plant‒microbe interactions. Research on pear-specific defense responses has enhanced our understanding of apoplastic interactions in nonmodel systems, with implications for fruit crop protection. A challenge in studying apoplast interactions in pear is the low abundance of apoplastic fluid in pear leaves compared with tobacco leaves. To address this, we optimized our protein extraction method to ensure high yield and purity. Using liquid chromatography‒tandem mass spectrometry (LC‒MS/MS) combined with RNA-sequencing (RNA-seq), we identified potential core apoplastic effectors secreted by *C. fructicola* as well as potential secretory resistance proteins in pear. Through this system, we identified a *C. fructicola* apoplastic effector, CfXyn11A, and its host target. CfXyn11A triggers an immune response in the nonhost *Nicotiana benthamiana* but evades detection in the host pear. Genetic and biochemical experiments revealed that CfXyn11A is a key effector essential for the pathogenicity and sugar uptake of *C. fructicola*. Immunoprecipitation coupled with mass spectrometry (IP‒MS) screening identified the pear xylanase inhibitor protein (PbXIP1), an aspartic protease-like protein, as the host target of CfXyn11A. This target enhances plant immunity by inhibiting the hydrolytic activity of CfXyn11A. This study provides a comprehensive characterization of the secretome during the interaction between pear and *C. fructicola*, offering new insights into pathogen‒host interactions in the apoplast.

## Results

### Extraction of apoplastic fluid from C. fructicola-infected pear leaves during the biotrophic stage

**Fig. 1:**
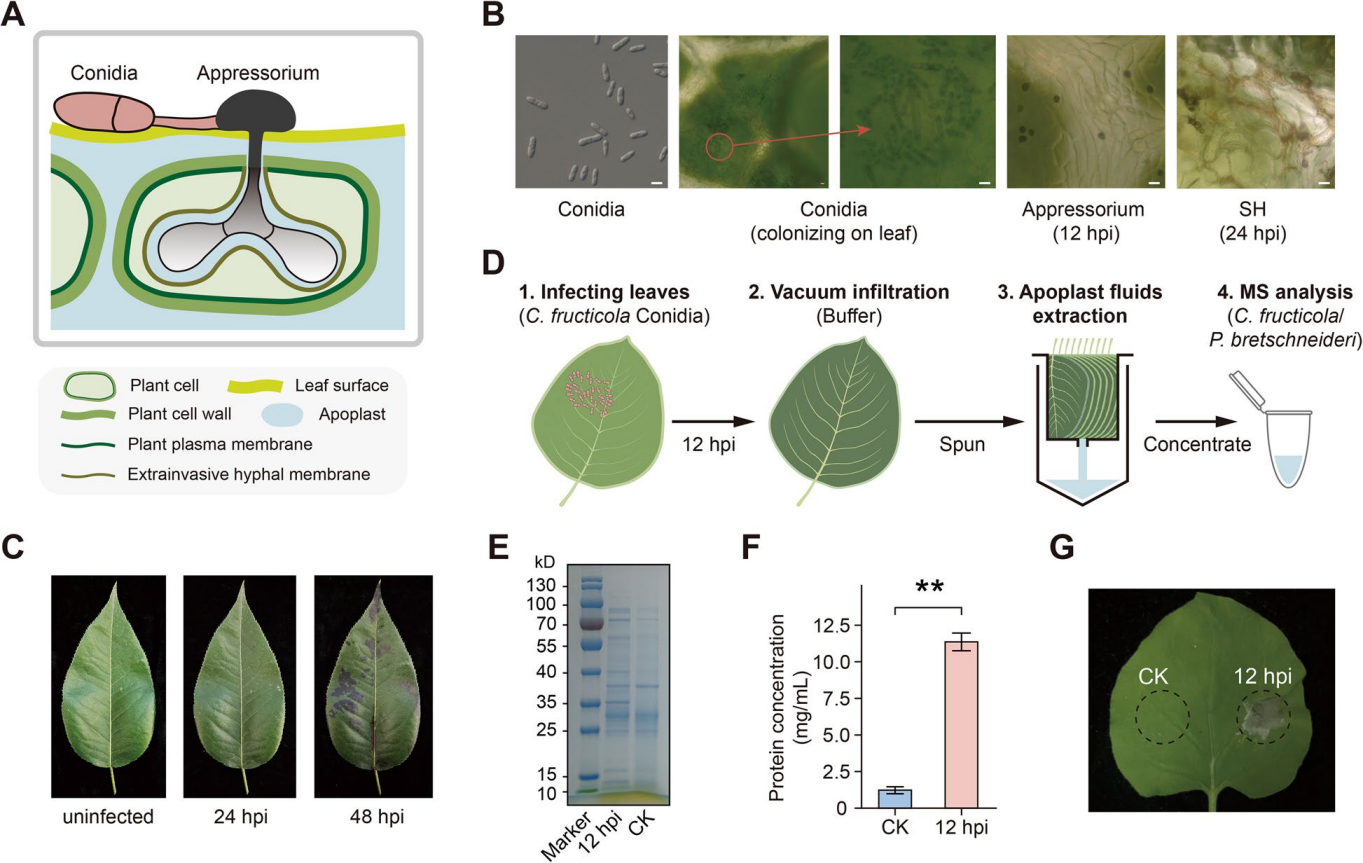
Collecting apoplast fluid from pear leaves after *C. fructicola* infection. **A** Illustration of the apoplast in a hemibiotrophic process during the infection of pear leaves by *C. fructicola*. Hemibiotrophic fungi *C. fructicola*. initially develop bulged biotrophic invasive hyphae, all intracellular structures are encased by the plant plasma membrane, known as the extrainvasive hyphal membrane (EHM). **B** Imaging of *C. fructicola* appressorial formation on pear leaf. Appressorium formation can be observed at 12 hpi, and secondary necrotrophic hyphae (SH) at 24 hpi. Bars: 10 μm. **C** Visible lesions can be observed at 48 hpi after *C. fructicola* infection. **D** Experimental design of pear leaf apoplast fuild extraction. Harvest intact pear leaves infected by *C. fructicola* infection at 12 hpi. Use vacuum infiltration to fill the leaves with buffer solution, then collect the infiltrate by centrifugation for LC–MS/MS identification. **E** SDS-PAGE of apoplast fluid from uninfected (CK) and infected leaves (12 hpi) with CBB. **F** Protein concentration of uninfected (CK) and infected leaves (12 hpi), values are means (± SEM) (*n* = 6). Asterisks indicate significant differences based on Student’s test (**P < 0.01). Experiments were repeated three times with similar results. **G** Inject apoplast fluid from uninfected (CK) and infected leaves (12 hpi) into tobacco plants, and take photos three days later

*C. fructicola* penetrates the plant tissue’s epidermis and cell wall using melanized appressoria (Fig. 1A, B) (Shang et al. 2020). After penetration, fungal hyphae grow intracellularly within the cell cavity without breaching the host protoplast (Liu et al. 2020; Shang et al. 2020). Appressoria typically appear 10–12 h post-inoculation (hpi). After establishment within one or more host cells, biotrophic intracellular hyphae subsequently develop secondary necrotrophic hyphae, leading to cell disruption observable as early as 24 hpi, although visible symptoms are not yet apparent at this stage (Fig. 1B, C). To prevent contamination from intracellular components, we inoculated ‘Cuiguan’ pear leaves with *C. fructicola* conidia and extracted the apoplastic fluid from pear leaves using a vacuum infiltration method at 12 hpi (Fig. 1D). The pear leaves were syringe-infiltrated with water, and apoplastic fluid was collected by centrifugation. After infiltration, the weight of pear leaves increased by an average of 19.38% compared with pre-infiltration weight (Fig. S1A, B). As centrifugation could not completely remove all liquid, the measured mass of the apoplastic fluid was less than the weight difference between infiltrated and pre-infiltrated pear leaves (Fig. S1C). Gel staining analysis revealed distinct protein compositions in apoplastic fluid collected from *C. fructicola*-infected leaves compared with uninfected control leaves (CK) (Fig. 1E), with protein concentration in infected leaves’ apoplastic fluid increasing up to tenfold compared with uninfected control leaves (Fig. 1F). Infiltration of apoplastic fluid from infected leaves into *N. benthamiana* leaves induced evident cell death within 2 days, while the apoplastic fluid from uninfected pear leaves failed to induce cell death (Fig. 1G).

### Identification of apoplastic proteins in C. fructicola-infected pear leaves

**Fig. 2:**
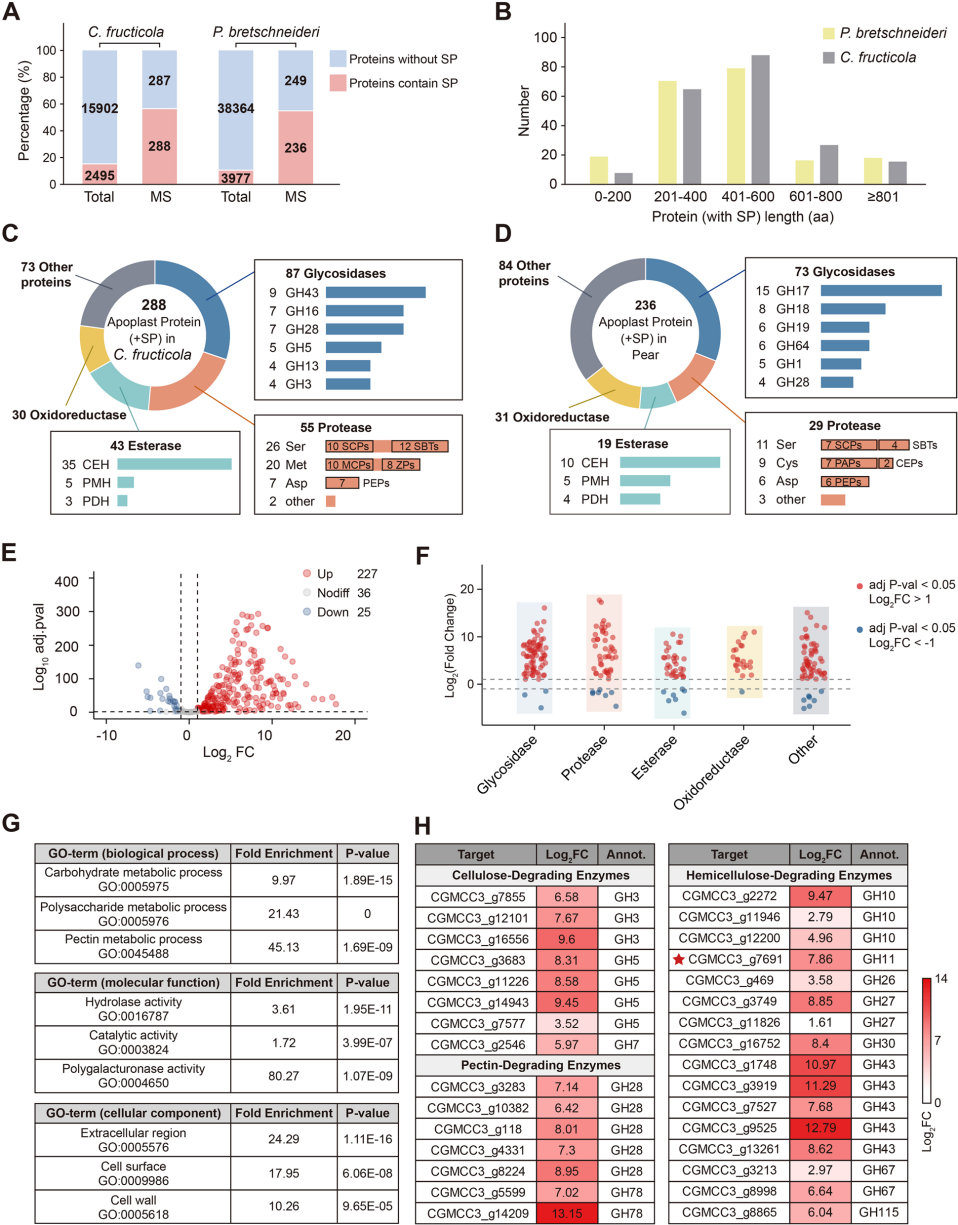
Composition of apoplastic proteins in pear leaves after *C. fructicola* infection. **A** The proportion of signal peptide-containing proteins in the apoplast fuild and the total proteome of both *C. fructicola* and pear. **B** The distribution of protein lengths in the apoplastic proteins. **C**, **D** Detected *C. fructicola* (**C**) and pear (**D**) apoplastic proteins predicted to have a signal peptide were annotated with PFAM and classified into glycosidases (blue), proteases (orange), lipases (green), oxidoreductase (yellow) and other protiens (grey), further subdivided into protein families. **E**, **F** The expression differences of *C. fructicola* apoplastic protein-coding genes at the conidia stage and 12 hpi (**E**), further subdivided into glycosidases, proteases, lipases, oxidoreductase and other protiens (**F**). **G** Significantly enriched GO terms in *C. fructicola* apoplastic proteins. **H** The log_2_-transformed fold change of CWDEs identified in *C. fructicola* apoplastic proteins. Data represent log_2_-transformed fold changes in gene expression (normalized against expression of the corresponding genes at the conidia stage)

To examine the composition of apoplastic proteins during the biotrophic stage of *C. fructicola* infection, we subjected the apoplastic fluid to trypsin digestion, analyzed it using LC‒MS/MS, and compared the protein profiles with databases of both Pear and *C. fructicola*. The analysis identified 1060 proteins, of which 524 were predicted to contain signal peptides (SPs) using SignalP (Armenteros et al. 2019). Specifically, 236 proteins were attributed to pear and 288 to *C. fructicola*. The proportion of SP-containing proteins in the apoplastic fluid significantly exceeded that in the entire genome (Fig. 2A; Tables S1 and S2). These proteins predominantly ranged from 200 to 600 amino acids in length (Fig. 2B). Comparison with the SUBA5 subcellular localization database for *Arabidopsis* proteins (v.5) revealed that 86.01% (203/236) of the predicted pear apoplastic proteins have homologs with extracellular or plasma membrane localization in *Arabidopsis*, including well-known plant secretory proteins such as chitinases and PR protein 1 (PR1) (Table S2). Pfam classification analysis of these 524 proteins showed that 51.27% (121/236) of the pear apoplastic proteins and 64.24% (185/288) of the *C. fructicola* apoplastic proteins were hydrolases (Fig. 2C, D; Tables S1 and S2). The remaining apoplastic proteins exhibited diversity, including numerous oxidoreductases (Fig. S2; Tables S1 and S2).

The 105 detected *C. fructicola* apoplastic hydrolases comprised 87 glycosidases, 55 proteases, and 43 esterases (Fig. 2C). The glycosidases belong to 40 distinct GH families, with the GH43 family being the most prevalent, playing a crucial role in plant cell wall degradation (Viborg et al. 2013). The apoplastic proteases identified were categorized into serine proteases (SEPs, 26 proteins), metalloproteinases (MPs, 20 proteins), aspartyl proteases (APs, 7 proteins), and 2 other proteases. The SEPs included 12 subtilisin-like proteases and 10 serine carboxypeptidase-like proteases. The MPs comprised 10 metallocarboxypeptidases and eight zinc peptidases. All seven APs were pepsin-like proteases. The esterases consisted of 35 carboxylic ester hydrolases, 5 phosphoric monoester hydrolases, and 3 phosphoric diester hydrolases. The composition of apoplastic hydrolases from pear aligned with previous reports in *A. thaliana* and *N. benthamiana* (Fig. 2D) (Goulet et al. 2010; Buscaill et al. 2019; Sueldo et al. 2024). The most abundant glycosidases were from the GH17 (15 proteins) and GH18 (8 proteins) families, which are thought to possess both glucanase and chitinase activities. In contrast to pathogens, pears lack MPs but secrete cysteine proteases, including 7 papain proteases and 2 cysteine endopeptidases, which are absent in *C. fructicola* apoplastic hydrolases.

During infection, a substantial proportion of the apoplastic proteins secreted by both *C. fructicola* and pear were predominantly hydrolases.

### C. fructicola apoplastic proteins exhibit widespread upregulation after infection

To identify the apoplastic effectors of *C. fructicola*, we collected samples of fresh *C. fructicola* conidia and pear leaves at 12 hpi. RNA from *C. fructicola* was extracted from infected leaf samples and subjected to transcriptome sequencing (Table S3). Analysis of gene expression patterns corresponding to apoplastic proteins detected by LC‒MS/MS revealed that a significant majority of the genes were upregulated (227/288), while only 25 were downregulated (Fig. 2E, F). Most genes encoding apoplastic proteins exhibited minimal expression during the conidium stage, with significant upregulation occurring only after pear leaf infection (Fig. 2F). Gene Ontology (GO) analyses of these upregulated genes indicated their primary association with extracellular regions, cell wall, and hydrolytic activity (Fig. 2G, Table S4). Among the identified *C. fructicola* apoplastic proteins, numerous CWDEs, including cellulose-, hemicellulose-, and pectin-degrading enzymes, showed upregulation (Fig. 2H).

### CfXyn11A belongs to the glycosyl hydrolase family 11 (GH11) and is commonly present as a multicopy gene in phytopathogenic fungi

**Fig. 3:**
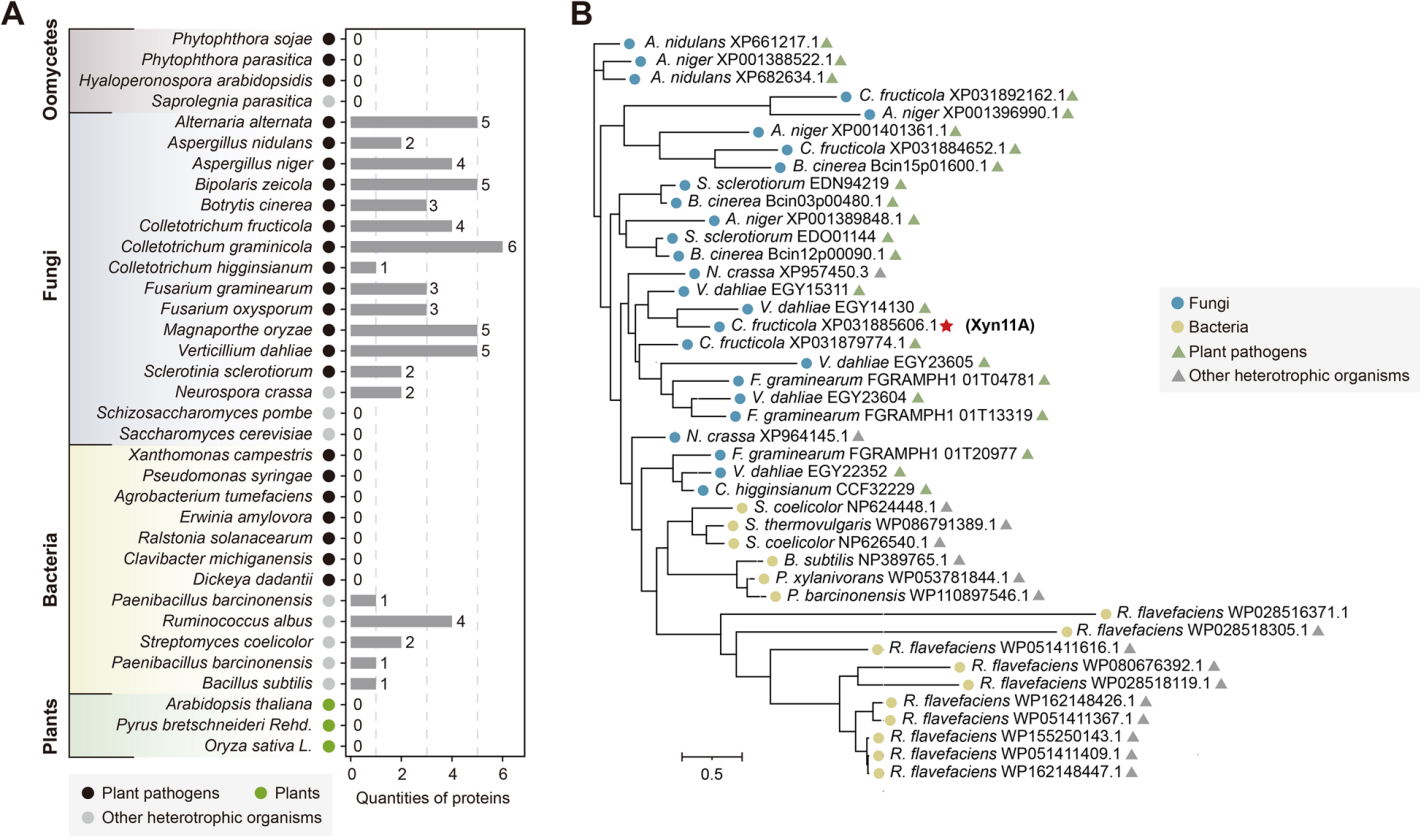
CfXyn11A belongs to the GH11 family and is frequently found as multicopy genes in plant pathogenic fungi. **A** The number of GH11 genes in different microbial or plant species. **B** Phylogenetic analysis of 42 GH11 genes from the indicated microbial species

Xylan, a principal hemicellulose component prevalent in plant cell walls, particularly in the secondary cell walls of woody plants, is essential for maintaining cell wall mechanical strength. Transcriptome analysis revealed significant upregulation of CGMCC3_g7691, annotated as endo-1,4-beta-xylanase, during infection (Fig. 2H). Functional domain analysis identified a SP and GH11 domain in CGMCC3_g7691; hence, it was named CfXyn11A. CfXyn11A is the sole GH11 member identified among *C. fructicola* apoplastic proteins. The GH11 family is characteristically associated with xylanase activity. Quantitative real-time PCR (qRT-PCR) analysis of CfXyn11A expression across various developmental and infection stages of *C. fructicola* revealed significant upregulation before 24 hpi (Fig. S3). This substantial expression increase suggests a potential role for CfXyn11A in *C. fructicola* pathogenicity. To assess the conservation of secreted CfXyn11A among microbial pathogens, we examined the genomes of 4 oomycete, 16 fungal, 12 bacterial, and 3 plant species. Sixty genes encoding homologous protein sequences were identified (Fig. 3A). These GH11 protein sequences are present in plant pathogenic fungi and some nonplant pathogenic bacteria but are absent in plants, plant pathogenic bacteria, plant pathogenic oomycetes, and yeast, indicating that GH11 proteins may be common in plant pathogenic fungi. Phylogenetic analysis revealed distinct clusters for GH11 proteins from plant pathogenic fungi and bacteria, indicating potential functional differences (Fig. 3B). Although multiple GH11 members are typically found in fungi (Watanabe et al. 2014), with 4 gene members usually present in *C. fructicola*, only CfXyn11A was identified in the LC‒MS/MS results of apoplastic fluid isolated from *C. fructicola*-infected pear leaves (Table S1). Transcriptome analysis revealed that the other 3 homologous genes were not expressed after infection, indicating that CfXyn11A might be the sole member involved in *C. fructicola* infection of pear.

### CfXyn11A is induced by xylan, contributes to the virulence of C. fructicola, and promotes extracellular xylan uptake

**Fig. 4:**
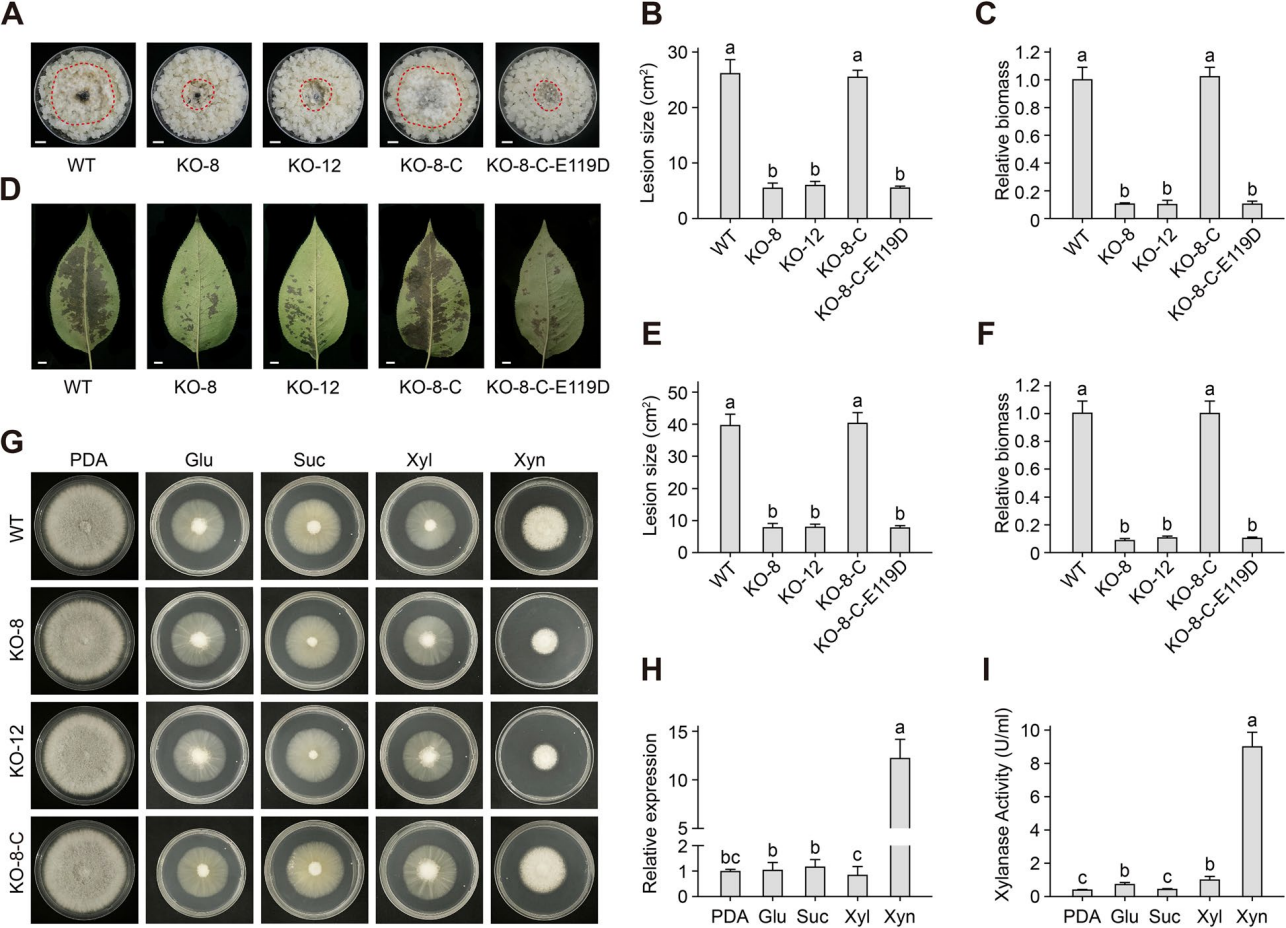
CfXyn11A contributes to the virulence of *C. fructicola* and promoting the uptake of extracellular xylan. **A-C** The deletion of CfXyn11A reduced the pathogenicity of *C. fructicola* on pear calli. One representative picture of pear calli inoculated with *C. fructicola* wild-type strain or mutants at 5 dpi is shown (**A**). The lesion areas (**B**) and relative biomass **(C)** of *C. fructicola* wild-type strain and mutants detected in the inoculated pear calli, values are means (± SEM) (*n* = 3). Different letters represent significant differences (*P* < 0.05; Duncan’s multiple range test). **D-F** The deletion of CfXyn11A significantly reduced the pathogenicity of *C. fructicola* on pear leaves. One representative picture of pear leaves inoculated with *C. fructicola* wild-type strain or mutants at 5 dpi is shown (**D**). The lesion areas (**E**) and relative biomass **(F**) of *C. fructicola* wild-type strain and mutants detected in the inoculated pear leaves, values are means (± SEM) (*n* = 3). Different letters represent significant differences (*P* < 0.05; Duncan’s multiple range test). **G** Mycelial growth of *C. fructicola* wild-type strain and mutants on medium supplemented with different sugars (glucose, sucrose, xylose and xylan) as the sole carbon source. **H** The expression level of CfXyn11A in *C. fructicola* on medium supplemented with different sugars as the sole carbon source, values are means (± SEM) (*n* = 3). Different letters represent significant differences (*P* < 0.05; Duncan’s multiple range test). **I** The xylanase activity in the fermentation supernatants of *C. fructicola* supplemented with different sugars as the sole carbon source, values are means (± SEM) (*n* = 3). Different letters represent significant differences (*P* < 0.05; Duncan’s multiple range test). **J**, **K** Effect of pH (**J**) and temperature (**K**) on xylanase activities of purified CfXyn11A. Values are means (± SEM) (*n* = 3)

To assess the role of CfXyn11A in *C. fructicola* virulence, we generated CfXyn11A deletion mutants using a split-marker approach (Fig. S4A). Potential CfXyn11A deletion mutants were identified by PCR assays using four pairs of detection primers (Fig. S4B). From these, KO-8 and KO-12 plants were randomly selected for further analysis. The deletion of CfXyn11A did not significantly influence the morphology or growth of *C. fructicola* on potato dextrose agar (PDA) plates (Fig. 4G). Conidia were inoculated on pear leaves and calluses, with the WT strain and complemented transformant KO-8-C serving as controls. The growth rates of the calli of the two CfXyn11A knockout mutants were significantly reduced compared with those of the WT and complemented transformants (Fig. 4A–C). Moreover, they exhibited more severe symptoms on pear leaves (Fig. 4D–F). These findings suggest that CfXyn11A plays a role in the virulence of *C. fructicola*.

To investigate the role of CfXyn11A in carbon source utilization, we cultivated the mutant and control strains in media containing various carbon sources. The results demonstrated that only when xylan served as the sole carbon source did the mutant and WT strains exhibit differential growth rates. The mutant strain displayed a significantly reduced growth rate compared with the WT strain (Fig. 4G). CfXyn11A expression was assessed by qRT-PCR, revealing that CfXyn11A transcript levels were the highest when xylan was the sole carbon source, with no significant induction observed under other conditions in the WT (Fig. 4H). To examine the effect on xylanase production, the WT strain was inoculated into liquid media and induced with different carbon sources. Enzyme activity peaked when xylan was the sole carbon source (Fig. 4I). These findings suggest that CfXyn11A expression is significantly regulated by the carbon source, with CfXyn11A production occurring exclusively in the presence of xylan as a carbon source.

### CfXyn11A targets the apoplast to elicit plant immune responses in N. benthamiana but evades host recognition

**Fig. 5:**
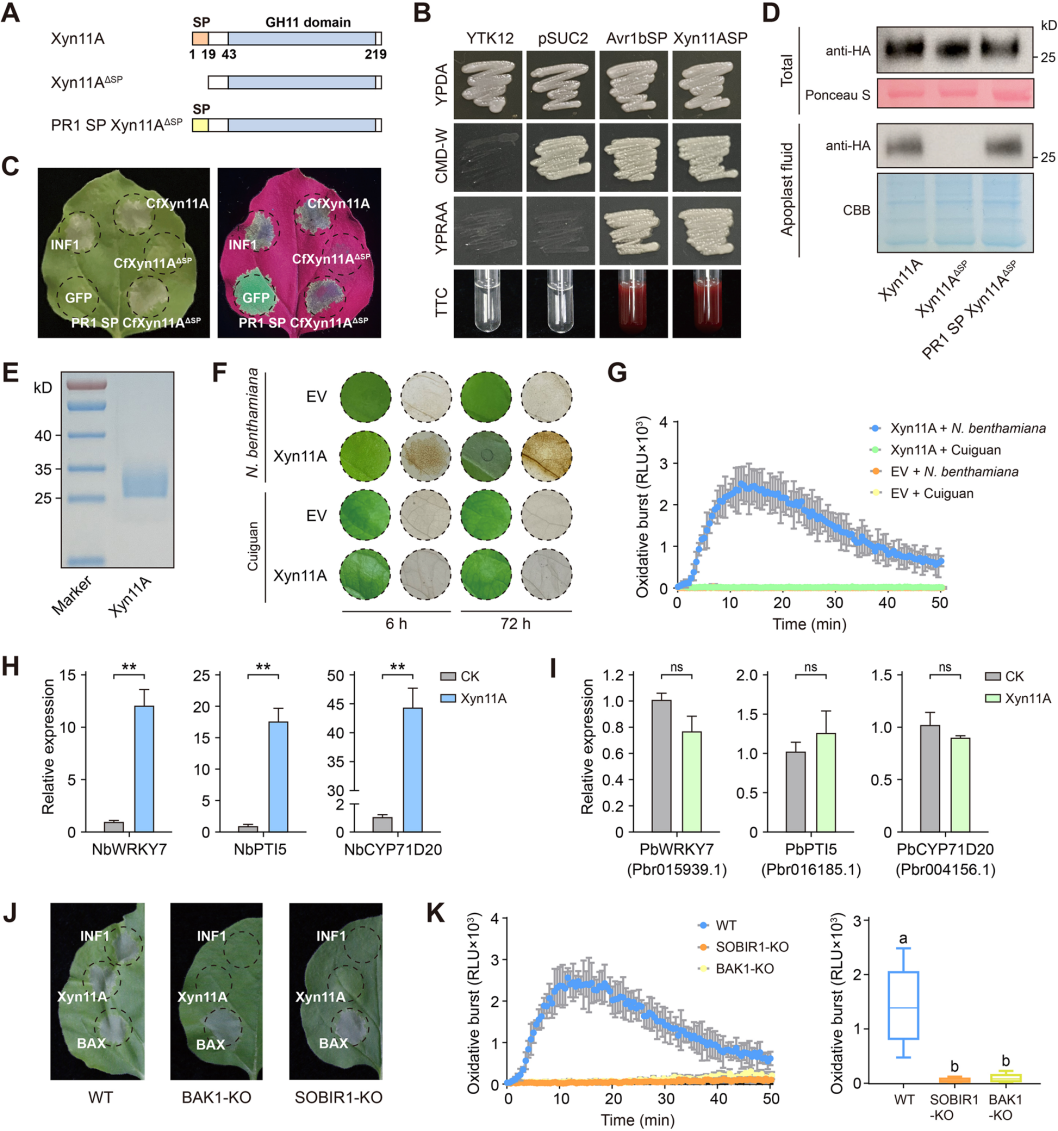
CfXyn11A can induce immunity in nonhost *N. benthamiana*, but cannot induce immune responses in its host, pear. **A** The schematic of CfXyn11A, signal peptide deletion mutant (CfXyn11A^ΔSP^) and the native signal peptide replaced by the signal peptide from PR1 (PR1-SP-CFXyn11A^ΔSP^). **B** Functional validation of CfXyn11A signal peptide. The strains were cultured on YPDA, CMD-W, or YPRAA medium for two days. Invertase enzymatic activity was assessed by converting TTC into insoluble red-colored TPF. **C** The cell death-inducing ability of CfXyn11A, CfXyn11A^ΔSP^ and PR1-SP-CFXyn11A^ΔSP^ in *N. benthamiana*, INF1 and green fluorescent protein (GFP) were used as positive and negative controls. All were transiently expressed in *N. benthamiana* leaves. Photographs were taken 5 days. **D** Detection of CfXyn11A, CfXyn11A^ΔSP^ and PR1-SP-CFXyn11A.^ΔSP^ in total protein and the apoplast fluid of *N. benthamiana* leaves. **E** SDS-PAGE of purified CfXyn11A from *Pichia pastoris* stained with Coomassie Brilliant Blue (CBB). **F** Leaves of *N. benthamiana* and pear infiltrated with EV and CfXyn11A (5 μM), stained by DAB staining. **G** Production of ROS in leaves of *N. benthamiana* and pear treated by 1 μM CfXyn11A. EV was used as a negative control. Values are means (± SEM) (*n* = 6). **H**, **I** Expression levels of immune marker genes in *N. benthamiana* (**H**) and pear (**I**) triggered by CfXyn11A, values are means (± SEM) (*n* = 3). Asterisks indicate significant differences based on Student’s test (**P < 0.01; ns, no significance). **J** The cell death-inducing ability of CfXyn11A, INF1 and BAX in *N. benthamiana* BAK1 and SOBIR1 knockout mutants. Photographs were taken 5 days. **K** Production of ROS in leaves of *N. benthamiana* BAK1 and SOBIR1 mutants treated by CfXyn11A (1 μM). Values are means (± SEM) (*n* = 6). Different letters represent significant differences (*P* < 0.05; Duncan’s multiple range test)

SP prediction indicated that CfXyn11A possessed an SP sequence comprising 19 amino acids (Fig. 5A). The secretory function of CfXyn11ASP was evaluated using a yeast signal trap assay system. Transformants containing CfXyn11ASP or the positive control Avr1bSP demonstrated growth on yeast extract-peptone-raffinose-antimycin agar (YPRAA) media with raffinose as the sole carbon source (Fig. 5B). These transformants also exhibited invertase secretion, as evidenced by forming the red product 1,3,5-triphenylformazan (TPF) (Fig. 5B).

To assess CfXyn11A’s role as an effector in the plant immune response, these proteins were transiently expressed in *N. benthamiana*. The complete coding sequences of these proteins were cloned and inserted into pBin, followed by agroinfiltration in *N. benthamiana,* utilizing green fluorescent protein (GFP) and INF1 as negative and positive controls, respectively. CfXyn11A demonstrated robust cell death-inducing activity in *N. benthamiana,* comparable to the positive control INF1 (Fig. 5C). To determine whether CfXyn11A requires targeting the apoplast to trigger cell death, we generated CfXyn11AΔSP by deleting the N-terminal SP, and created PR1-SP-CfXyn11A^ΔSP^ by adding the SP from PR1 protein (Fig. 5A). CfXyn11A^ΔSP^ expression did not induce cell death in *N. benthamiana*, whereas PR1-SP-CfXyn11A^ΔSP^ expression successfully triggered cell death (Fig. 5C). Total protein and apoplastic mixture were extracted from the leaves of transiently transformed *N. benthamiana* plants. Notably, CfXyn11A and PR1-SP-CfXyn11A^ΔSP^ were detectable in both apoplastic fluid and total protein extracts; however, CfXyn11A^ΔSP^ was not detected in the apoplastic fluid (Fig. 5D). These findings suggest that CfXyn11A must be targeted to the extracellular space of *N. benthamiana* tissue to effectively induce cell death.

To elucidate the function of CfXyn11A, the protein was expressed and purified from *Pichia pastoris* (Fig. 5E). The purified CfXyn11A induced cell death and ROS accumulation in *N. benthamiana* (Fig. 5F), while the empty vector (EV) control did not elicit these responses. Notably, CfXyn11A failed to trigger cell death or ROS accumulation in its host, pear. To assess CfXyn11A’s ability to elicit plant immune responses, we treated *N. benthamiana* and pear leaf disks with the purified protein and monitored ROS burst using a luminol assay. CfXyn11A induced a ROS burst in *N. benthamiana*, similar to some reported PAMPs, but pear leaves remained unresponsive (Fig. 5G). In addition, qRT-PCR analysis revealed that CfXyn11A upregulated PAMP-responsive genes WRKY7, Pti5, and CYP71D20 in *N. benthamiana* (Fig. 5H), while defense genes in pear were not induced (Fig. 5I). These pear defense genes, homologous to WRKY7, Pti5, and CYP71D20 in *N. benthamiana*, were significantly upregulated after infection with *C. fructicola* or *Erwinia amylovora* (Han et al. 2021, 2024a). To investigate the host specificity of CfXyn11A, we infiltrated CfXyn11Arec into expanded leaves of various plant species. CfXyn11Arec induced cell death in soybean, tomato, potato, and rice, but not in pepper, corn, and three other pear varieties (Fig. S5A). The luminol assay confirmed that CfXyn11A did not induce ROS burst in these four pear varieties (Fig. S5B), whereas the positive control flg22 did (Fig. S5C). These findings suggest that the immune-activating capacity of CfXyn11A is non-universal.

*C. fructicola* is a nonadapted pathogen of *N. benthamiana* that induces nonhost resistance (NHR) in *N. benthamiana* (Han et al. 2024b). We inoculated *N. benthamiana* with a conidial suspension of *C. fructicola* and CfXyn11Arec, followed by inoculation with *P. capsici*. Compared with the control group, the number of pathogen-induced lesions on leaves injected with the spore suspension and CfXyn11Arec was significantly reduced (Fig. S6). However, this involvement is limited, as the CfXyn11A mutant also failed to effectively infect *N. benthamiana* (Fig. S7). BAK1 and SOBIR1 are two central receptor-like kinases that participate in multiple leucine-rich repeat-type PRR-mediated immune signal transduction pathways (Liebrand et al. 2014). To elucidate the molecular mechanism underlying the activation of defense signaling, we assessed CfXyn11A-triggered immune responses in BAK1- and SOBIR1-knockout *N. benthamiana* mutants. The expression of CfXyn11A and INF1 did not induce cell death in the BAK1 and SOBIR1 knockout mutants, while the positive control BAX maintained its ability to induce cell death (Fig. 5J). The CfXyn11A-triggered ROS burst was also significantly lower in the BAK1 and SOBIR1 knockout mutants compared with WT *N. benthamiana* (Fig. 5K)*.* Collectively, these findings suggest that CfXyn11A contributes to NHR in *N. benthamiana* against *C. fructicola* while evading detection by the pear immune system.

### Enzyme activity of CfXyn11A is not required for elicitor activity

**Fig. 6:**
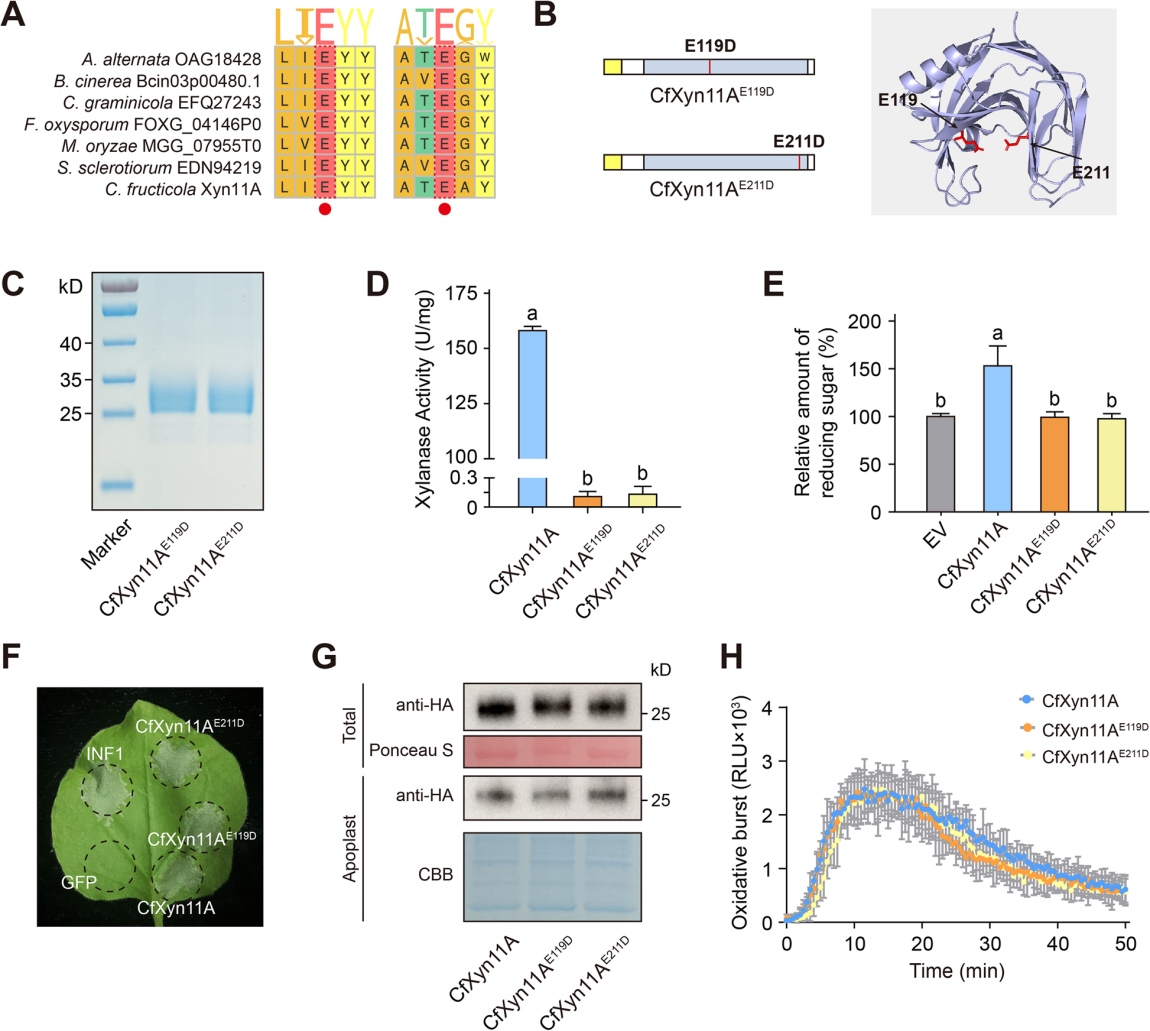
The cell death-inducing ability by CfXyn11A is independent of its enzymatic activity. **A** Multiple sequence alignment of CfXyn11A other reported GH11 family members, where the predicted enzyme active sites are indicated by boxes. **B** The prediction of the 3D structure of CfXyn11A, with the enzyme active sites highlighted in red. **C** SDS-PAGE of purified CfXyn11A^E119D^ and CfXyn11A^E211D^ from *Pichia pastoris* stained with CBB. **D** Xylanase Activity of wild-type and Mutants, values are means (± SEM) (*n* = 3). Different letters represent significant differences (*P* < 0.05; Duncan’s multiple range test). **E** The reducing sugar levels in apoplast fluid of *N. benthamiana* transiently expressing CfXyn11A, CfXyn11A^E119D^ and CfXyn11A^E211D^, values are means (± SEM) (*n* = 3). Different letters represent significant differences (*P* < 0.05; Duncan’s multiple range test). **F** The cell death-inducing ability of CfXyn11A^E119D^ and CfXyn11A^E211D^ in *N. Benthamian*, INF1 and GFP were used as positive and negative controls. All were transiently expressed in *N. benthamiana* leaves. Photographs were taken 5 days. **G** Detection of CfXyn11A, CfXyn11A^E119D^ and CfXyn11A^E211D^ in total protein and the apoplast fluid of *N. benthamiana* leaves. **H** Production of ROS in leaves of *N. benthamiana* treated by CfXyn11A, CfXyn11A^E119D^ and CfXyn11A.^E211D^ (1 μM), values are means (± SEM) (*n* = 6)

GH11 proteins are widely used in industrial applications for converting xylan into economically valuable products such as xylooligosaccharides, xylose, xylitol, and ethanol, owing to their xylanase activity (Rahmani et al. 2019). Multiple sequence alignment analysis revealed that CfXyn11A contains two conserved catalytic residues, Glu-119 and Glu-211, similar to GH11 members from other microorganisms (Fig. 6A). To determine their contribution to hydrolase activity, we substituted Glu-119 and Glu-211 individually with aspartate by site-directed mutagenesis (Fig. 6B) and expressed the mutant proteins (CfXyn11A^E119D^ and CfXyn11A^E211D^) in *P. pastoris* (Fig. 6C). The xylanase activity of CfXyn11A, CfXyn11A^E119D^, and CfXyn11A^E211D^ were assessed using the 3,5-dinitrosalicylic acid (DNS) assay. While CfXyn11A demonstrated xylanase activity, the activity of CfXyn11A^E119D^ and CfXyn11A^E211D^ was eliminated (Fig. 6D). The virulence of the CfXyn11A deletion mutant was not restored by transformation with the site-directed mutant KO-8-C-E119D (Fig. 4A, B), suggesting that the xylanase activity of CfXyn11A plays a crucial role in the virulence of *C. fructicola*. The optimum temperature for CfXyn11A activity was 40–50 °C at pH 4.0–6.0 (Fig. S8), which may facilitate its adaptation to the acidic environment within plants.

The apoplastic fluid of *N. benthamiana* leaves transiently expressing CfXyn11A exhibited elevated levels of reducing sugars, whereas no increase was observed in samples containing CfXyn11A^E119D^ and CfXyn11A^E211D^ (Fig. 6E). Transient expression of these two mutant proteins demonstrated a cell death-inducing capacity comparable to that of CfXyn11A (Fig. 6F). Immunoblot analysis confirmed protein production (Fig. 6G). CfXyn11A^E119D^ and CfXyn11A^E211D^ elicited ROS burst patterns in *N. benthamiana* similar to those induced by CfXyn11A (Fig. 6H). These findings suggest that the enzyme activity of CfXyn11A is not essential for its capacity to elicit plant immune responses.

### Pear apoplastic protein PbXIP1 binds to CfXyn11A and inhibits its enzyme activity

**Fig. 7:**
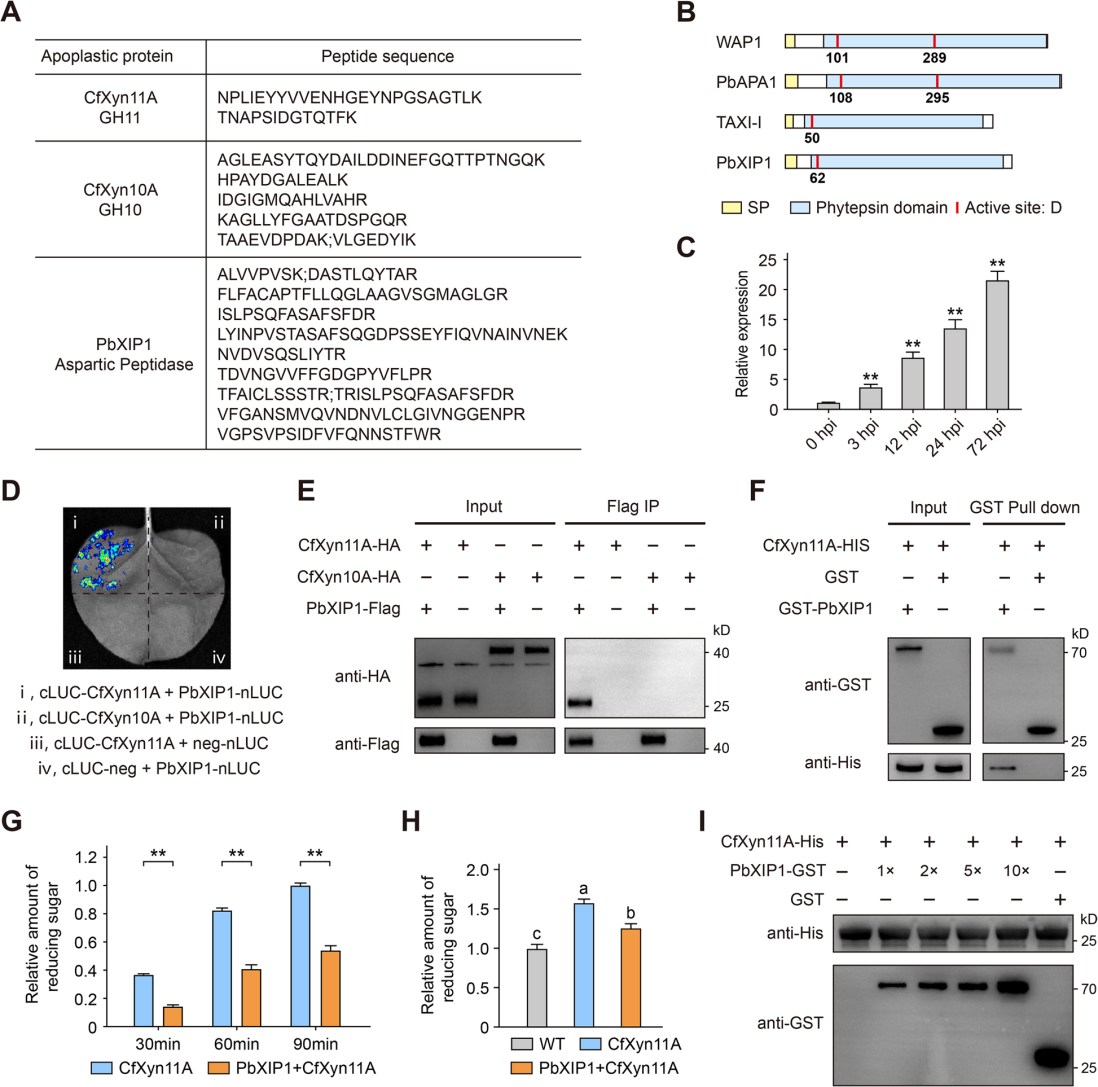
PbXIP1 physically interacts with CfXyn11A in vitro and in vivo and inhibits its activity. **A** Matching peptide was detected of CfXyn11A, CfXyn10A and PbXIP1 in apoplast fluid. **B** Predicted Pfam domain and catalytic aspartate residue of WPA1, PbAPA1, TAXI-1 and PbXIP1. **C** The expression pattern of PbXIP1 at multiple time points after pear infection with *C. fructicola,* values are means (± SEM) (*n* = 3). Asterisks indicate significant differences based on Student’s test (**P < 0.01). **D** Luciferase complementation imaging (LCI) assay was performed to test the interaction of CfXyn11A and CfXyn10A with PbXIP1 in vivo. **E** Co-immunoprecipitation (IP) assay was performed to test the interaction of CfXyn11A and CfXyn10A with pear PbXIP1 in vivo. HA-tagged CfXyn11A or HA-tagged CfXyn10A transiently co-expressed with Flag-tagged CfXyn11A in *N. benthamiana* leaves. IP was performed using antiFlag affinity gel, followed by western blot analysis using HA or Flag antibody. **F** GST pull-down assay was performed to test the interaction between CfXyn11A and PbXIP1 in vitro. His-tagged CfXyn11A were mixed with GST-tagged PbXIP1 or GST and passed through anti-GST agarose-conjugated beads, followed by western blot analysis using His or GST antibody. **G** The reducing sugars released by CfXyn11A (1 µg) in vitro in the presence of PbXIP1 (5 µg), values are means (± SEM) (*n* = 3). Asterisks indicate significant differences based on Student’s test (**P < 0.01). **H** The reducing sugar levels in apoplast fluid of *N. benthamiana* transiently expressing CfXyn11A and PbXIP1, values are means (± SEM) (*n* = 3). Different letters represent significant differences (*P* < 0.05; Duncan’s multiple range test). **I** Detection of the degradation of PbXIP1 on CfXyn11A in vitro. Purified CfXyn11A was incubation with PbXIP1-GST or GST incubated at 25 °C for 6 h. The PbXIP1 and CfXyn11A were analyzed by immunoblotting with anti-His and anti-GST antibodies

During infection, pears secrete numerous proteins into the apoplast space (Fig. 2). To identify potential host inhibitors of CfXyn11A, we used IP–MS to screen for CfXyn11A-binding proteins. By comparing results from *N. benthamiana* with identified host apoplastic proteins, we discovered a protein sharing 30% amino acid homology with *Triticum aestivum* xylanase-inhibitor (TAXI), which we designated as PbXIP1 (Fig. 7A, Table S5). This was the sole identified member of the xylanase-inhibitor family. TAXI inhibits *Aspergillus niger* xylanase, reducing bread volume and impacting wheat bread production (Debyser et al. 1999). Similar to TAXI, PbXIP1 resembles aspartate proteases but lacks a critical catalytic aspartate residue and exhibits no protease activity (Fig. 7B). To confirm PbXIP1’s secretion capability, we performed a secretion assay in yeast, verifying the secretion ability of PbXIP1-SP (Fig. S9). qRT-PCR analysis revealed significant upregulation of PbXIP1 following *C. fructicola* infection (Fig. 7C)*.* The interaction between CfXyn11A and PbXIP1 was confirmed by a luciferase complementation imaging (LCI) assay in *N. benthamiana* (Fig. 7D). A co-IP assay demonstrated CfXyn11A’s association with PbXIP1 in *N. benthamiana* (Fig. 7E). Glutathione S-transferase (GST) pull-down data indicated a direct interaction between CfXyn11A and PbXIP1 in vitro (Fig. 7F). Coexpression of CfXyn11A and PbXIP1 in *N. benthamiana* leaves showed that PbXIP1 does not affect CfXyn11A’s ability to induce cell death in *N. benthamiana* leaves (Fig. S10). By monitoring reducing sugar release, we found that PbXIP1 significantly inhibited CfXyn11A-catalyzed xylan depolymerization (Fig. 7G, H). To ensure that CfXyn11A’s reduced catalytic activity was not due to protein degradation, CfXyn11A was incubated with varying PbXIP1 concentrations for 6 h, confirming CfXyn11A’s stability (Fig. 7I). To determine which amino acids mediate CfXyn11A binding to PbXIP1, we used AlphaFold to predict CfXyn11A and PbXIP1 structures (Jumper et al. 2021) and used GRAMM to model protein interactions (Singh et al. 2024). The model suggests that PbXIP1 binds CfXyn11A at four regions (G1–G4) (Fig. S11A). Alanine substitution mutations in each region were tested to evaluate their ability to disrupt the PbXIP1–CfXyn11A interaction (Fig. S11B). Co-IP assays in plants revealed that mutations in the G4 region (covering nine amino acids of PbXIP1, positions 424–432) almost completely abolished CfXyn11A interaction. In addition, G1 (positions 205–209) and G2 (positions 226–234) were required for full binding activity. Furthermore, we tested whether CfXyn11A’s enzyme active site affects PbXIP1 binding. Mutations in the enzyme active site did not impact CfXyn11A’s full binding activity with PbXIP1 (Fig. S11C). Among *C. fructicola*’s apoplastic proteins, besides CfXyn11A, another xylanase belonging to the GH10 family was discovered and named CfXyn10A (Fig. 7A). However, CfXyn10A did not interact with PbXIP1 in vivo (Fig. 7D, E). The inhibition of CfXyn11A’s xylanase activity by PbXIP1 was specific; thus, PbXIP1 is not a broad-spectrum xylanase inhibitor.

### PbXIP1 positively regulates resistance to C. fructicola in pear

**Fig. 8:**
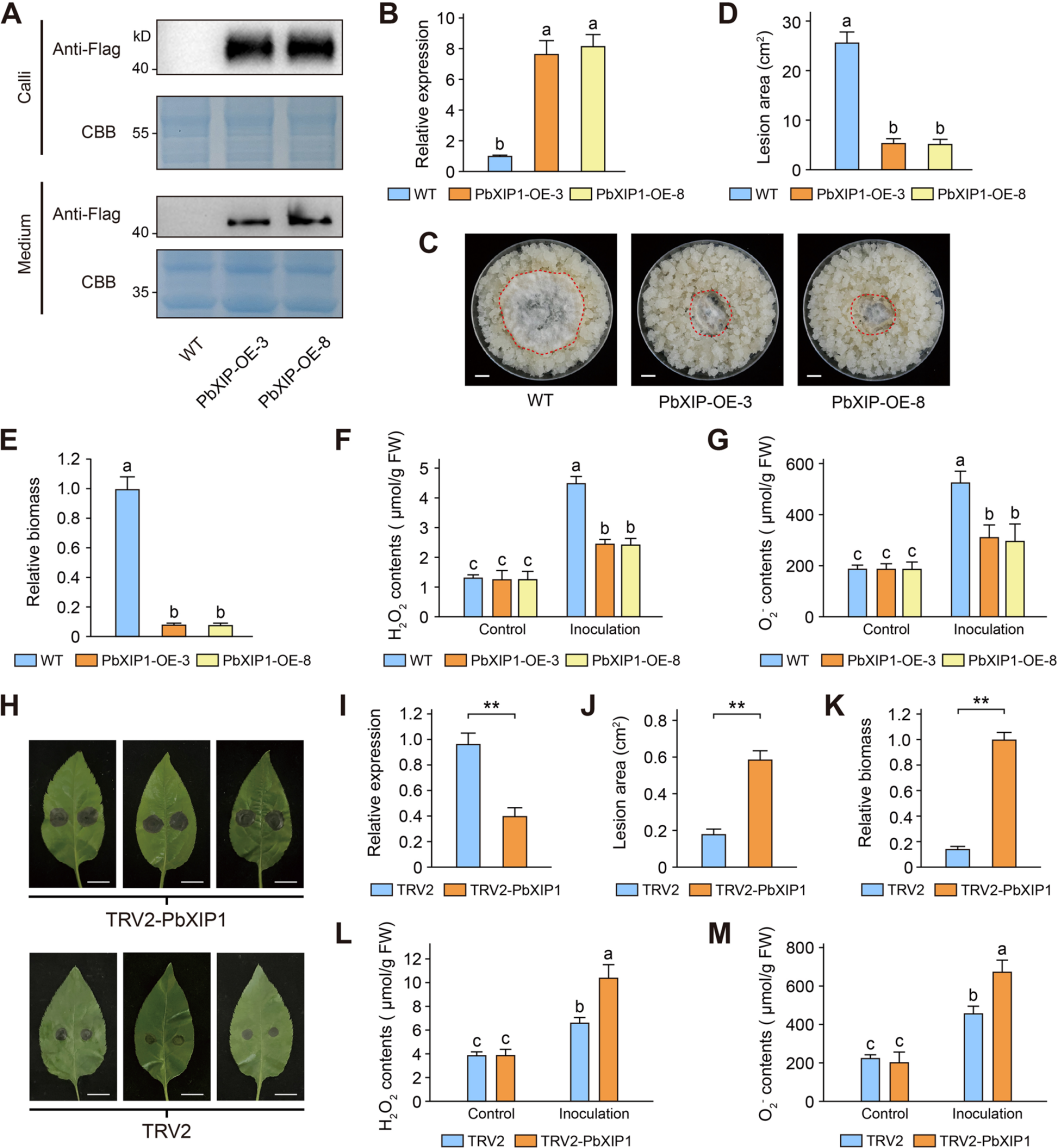
PbXIP1 positively regulates pear resistance against *C. fructicola*. **A-G** Overexpression of PbXIP1 enhanced resistance to *C. fructicola* in pear calli. **A** PbXIP1 was identified in the nutrient medium for the suspension culture of calli and callus tissue protein by immunoblotting with anti-Flag. **B** The expression level of PbXIP1 in wild-type calli and transgenic calli, values are means (± SEM) (*n* = 3). Different letters represent significant differences (*P* < 0.05; Duncan’s multiple range test). One representative picture of wild-type callus and transgenic callus inoculated with *C. fructicola* at 5 dpi is shown (**C**). The lesion areas (**D**) and relative biomass (**E**) of *C. fructicola* detected in the inoculated calli, values are means (± SEM) (*n* = 3). Different letters represent significant differences (*P* < 0.05; Duncan’s multiple range test). The H_2_O_2_ (**F**) and O_2_^−^ (**G**) levels were detected in WT and transgenic calli at 5 dpi, values are means (± SEM) (*n* = 3). Different letters represent significant differences (*P* < 0.05; Duncan’s multiple range test). (**H-M**) Silencing of PbXIP1 resulted in reduced resistance to *C. fructicola.* (**I**) PbXIP1 expression levels after VIGS treatment, values are means (± SEM) (*n* = 3). Asterisks indicate significant differences based on Student’s test (**P < 0.01). (**H**) Phenotype of TRV2 and TRV2-PbXIP1 plants inoculated with *C. fructicola* at 3 dpi. The lesion areas (**J**) and relative biomass (**K**) of *C. fructicola* detected in the TRV2 and TRV2-PbXIP1 plants, values are means (± SEM) (*n* = 3). Asterisks indicate significant differences based on Student’s test (**P < 0.01). The H_2_O_2_ (**L**) and O_2_.^−^ (**M**) levels were detected in TRV2 and TRV2-PbXIP1 plants at 5 dpi, values are means (± SEM) (*n* = 3). Different letters represent significant differences (*P* < 0.05; Duncan’s multiple range test)

To investigate the role of CfXyn11A in pear resistance against *C. fructicola*, we used the pCAMBIA2300-PbXIP1-Flag vector for stable PbXIP1 overexpression in pear calli by *Agrobacterium*-mediated transformation. Confirmation of PbXIP1 overexpression was achieved by qRT-PCR and Western blot analyses. The transgenic lines exhibited significantly higher PbXIP1 expression levels compared with WT calli, with PbXIP1-Flag protein detected in both transgenic lines (Fig. 8A, B). Furthermore, PbXIP1 was observed in the culture supernatant after 25 days of pear callus suspension culture (Fig. 8A). Subsequently, we inoculated the calli with *C. fructicola* to assess changes in resistance after PbXIP1 overexpression. At 5 days post-inoculation, the growth of *C. fructicola* was slower in PbXIP1-overexpressing calli than in WT calli (Fig. 8C–E). Under standard conditions, H_2_O_2_ and O_2_^−^ levels showed no significant difference between WT and transgenic calli. However, upon *C. fructicola* inoculation, the reduced fungal mass in the PbXIP1 transgenic calli lines resulted in significantly lower levels of H_2_O_2_ and O_2_^−^ compared with WT calli (Fig. 8F, G).

To investigate the role of PbXIP1 in pear resistance to *C. fructicola*, virus-induced gene silencing (VIGS) was used to downregulate PbXIP1 expression in pear leaves. qRT-PCR analysis revealed that PbXIP1 expression in tobacco rattle virus 2 (TRV2)-PbXIP1 leaves was approximately one-third of that in TRV2 leaves (Fig. 8I), confirming successful silencing of PbXIP1. After inoculation with *C. fructicola*, the disease spot area in TRV2-PbXIP1 leaves was more than three times larger than that in TRV2 leaves (Fig. 8H, J)*.* In addition, TRV2-PbXIP1 leaves exhibited increased fungal mass and elevated levels of H_2_O_2_ and O_2_^−^ (Fig. 8K–M). These findings suggest that PbXIP1 plays a role in enhancing pear resistance to *C. fructicola*.

## Discussion

### Hemibiotrophic stage and protein secretion analysis

During the initial infection stage of *C. fructicola*, conidia develop melanized appressoria and specialized infection vesicles, forming biotrophic intracellular hyphae within living host cells enveloped by an intact host plasma membrane (Gan et al. 2013; Shang et al. 2020). This phase is referred to as the hemibiotrophic stage. By leveraging this characteristic of *C. fructicola*, we can find a representative of proteins secreted by both host and pathogen, thereby minimizing contamination from intracellular components due to cell death. The identification of pathogenic apoplastic proteins typically involves the isolation of microbial culture supernatants or prediction based on SPs and transmembrane domains, methods that have led to the identification of several potent elicitors (Ma et al. 2015; Chen et al. 2022; Han et al. 2024b). However, certain apoplastic effectors induced solely during plant‒pathogen interactions remain challenging to detect. In our previous research, we extracted apoplastic fluid components from infected pear hosts using a vacuum infiltration method, successfully identifying several apoplastic resistance proteins (Han et al. 2024a). The present study optimized inoculation and separation methods, successfully isolated apoplastic fluid from *C. fructicola*–pear interactions, and established a repository of *C. fructicola*–pear apoplastic proteins.

### Identification of apoplastic effectors

Unlike conventional PAMPs that elicit immune responses, immune reactions triggered by proteinaceous apoplastic effectors often result in extensive cell death. PCD is commonly linked to plant defense responses against abiotic and biotic stresses (Niu et al. 2024). Hence, the *N. benthamiana* system provides an effective method for screening effectors capable of inducing plant cell death (Cao et al. 2024; Han et al. 2024b). We identified the GH11 CfXyn11A, recognized as a PAMP by the nonhost *N. benthamiana*, which plays a crucial role in *C. fructicola* pathogenicity. CfXyn11A-induced cell death in *N. benthamiana* required its secretion into the apoplast and the involvement of BAK1 and SOBIR1 (Fig. 5). Notably, the substitution of two conserved glutamate residues eliminated xylanase activity without affecting its elicitor function. These findings strongly indicate that plants perceive CfXyn11A through the membrane-localized PRR–BAK1 complex. NHR, considered the most durable and efficient plant immune system, typically manifests as cell death at infection sites in nonhost plants when challenged by nonadapted pathogens, halting pathogen growth at an early stage (Nurnberger et al. 2005; Ayliffe et al. 2019). *C. fructicola* is a nonadapted pathogen of *N. benthamiana* (Han et al. 2024b). Our results demonstrate that *N. benthamiana* leaves treated with either CfXyn11A or conidial suspensions exhibited strong resistance, indicating CfXyn11A’s potential involvement in triggering NHR formation in *N. benthamiana* (Fig. S6). ROS have been widely reported to participate in various physiological processes and resistance responses in pear (Wu et al. 2023; Zhang et al. 2024). Interestingly, CfXyn11A failed to trigger ROS, or defense gene expression in the host pear. These observations suggest that the host pear does not effectively recognize CfXyn11A as a PAMP or immune elicitor. The pear immune system may require additional cues, such as specific coreceptors or signaling molecules, to recognize CfXyn11A as a threat. Alternatively, CfXyn11A may have developed mechanisms to evade detection by the pear immune system, enabling pathogen establishment without eliciting a robust host defense response. This underscores the complexity of plant‒pathogen interactions, where pathogen effectors can modulate host immunity, and highlights the need for further investigation into the molecular mechanisms underlying host recognition and immune response activation in pear.

### Role of xylanases in pathogen infection

The plant apoplast is a sugar-rich environment, and effective sugar uptake is crucial for pathogen infection (Naseem et al. 2017). Xylans, the primary components of hemicellulose in cell walls, constitute 15–30% of the cell wall content in angiosperm hardwoods (Singh et al. 2003). These polysaccharides consist of β-1,4-linked xylose residues with various side branch substituents (Kulkarni et al. 1999). Currently identified hydrolases with xylanase activity include glycoside hydrolase families 5, 7, 8, 10, 11, and 43 (Collins et al. 2005). However, except for GH10 and GH11, other GHs demonstrate low xylanase activity (Collins et al. 2005). GH11 is the only monospecific xylanase that acts exclusively on D-xylose-containing substrates (Biely et al. 1997). GH11 was found to be more effective at hydrolyzing wheat bran compared with GH10 (Beaugrand et al. 2004). While the strain *C. gloeosporioides* PENZ, part of the *C. gloeosporioides* species complex, was unable to survive on a medium with xylose as the sole carbon source (Madan et al. 1994), our study revealed that *C. fructicola* exhibited robust growth on xylose. Research on the carbon source selectivity of *C. fructicola* is limited; however, genome analysis indicates that *C. fructicola* possesses a complete xylose reduction–oxidation pathway, including xylulose kinase, which is necessary for growth in culture media with D-xylulose as the sole carbon source (Rodriguez-Pena et al. 1998; Armitage et al. 2020). In this study, genetic and biological experiments demonstrated that CfXyn11A can be induced by xylan and converts extracellular xylan into absorbable monosaccharides, contributing to the virulence of *C. fructicola*. The complete hydrolysis of xylan requires the cooperative action of multiple hydrolases, which may explain why the CfXyn11A mutant strain has not entirely lost its virulence and can still grow slowly on a medium where xylan is the sole carbon source (Fig. 4).

### Xylanase inhibitors and plant defense

Microbial xylanases are extensively utilized in the food industry, and xylanase inhibitors, which were first discovered in wheat proteins, influence breadmaking quality (Debyser et al. 1999; Weng et al. 2010). Limited research exists on these inhibitors’ role in plant defense, primarily due to their exceptional specificity (Jashni et al. 2015). The combination of IP–MS and LC‒MS/MS offers an approach for investigating enzyme–inhibitor interactions in nonmodel plant apoplasts. Using this method, we identified PbXIP1, an inhibitor of CfXyn11A, in the host plant. Upon infection, PbXIP1 is strongly upregulated and secreted into the apoplast, where it significantly inhibits CfXyn11A activity and enhances pear disease resistance (Figs. 7 and 8). Soybean secretes GmGIP1, which binds to the xyloglucan-specific endoglucanase PsXEG1 of *P. sojae* and inhibits its activity (Ma et al. 2017). PsXEG1 belongs to the GH12 family and is structurally related to GH11, but GIP cannot inhibit proteins from the GH11 family (Yoshizawa et al. 2011; Tundo et al. 2022). Our findings indicate that PbXIP1 inhibits the hydrolytic activity of CfXyn11A independently of enzyme activity. PbXIP1’s inhibitory function occurs by interactions with CfXyn11A at specific surface regions rather than by broad, nonspecific mechanisms (Figs. 7 and S9). However, these interactions represent only a small fraction of the complex interplay, as *C. fructicola* secretes over 200 apoplastic proteins, many of which are hydrolases (Fig. 2). The abundance and diversity of these proteins indicate that further investigation into their roles could uncover novel pathogenicity mechanisms. A promising direction for future research may be the development of high-throughput screening methods to identify enzyme‒inhibitor interactions, which could become a focal point in the study of plant apoplast-based disease resistance.

### Proposed working model

**Fig. 9:**
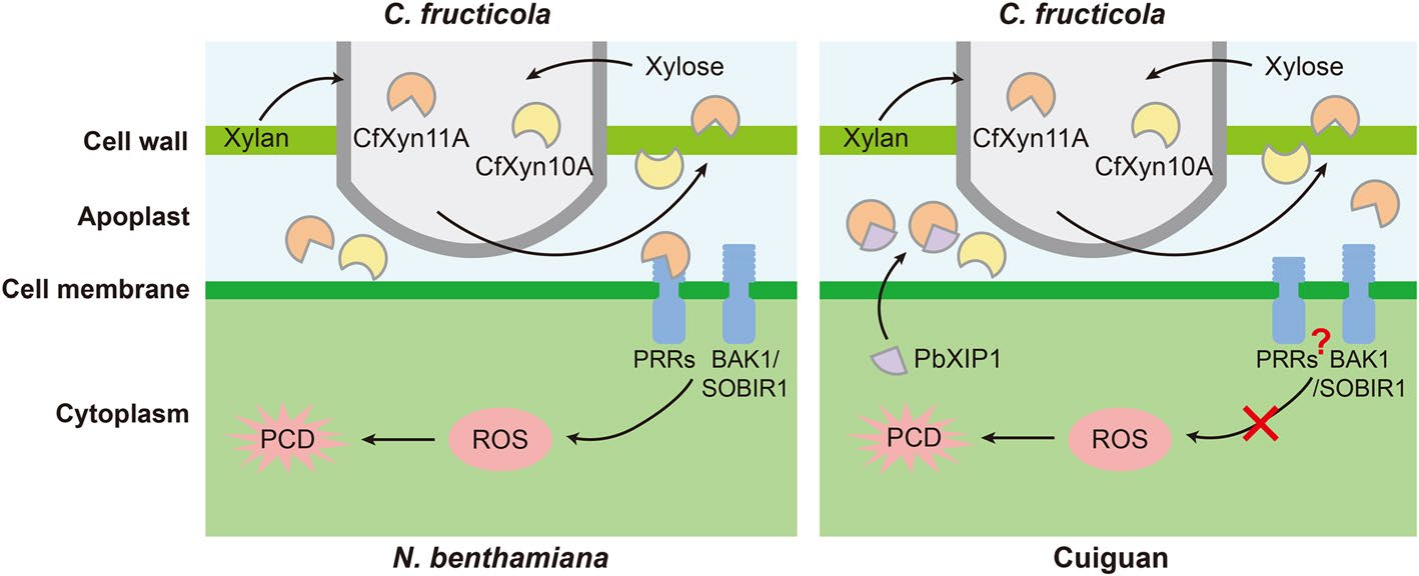
A proposed model illustrating CfXyn11A-mediated virulence and PbXIP1-promoted resistance. Upon sensing xylan components in the cell wall, CfXyn11A in *C. fructicola* is induced, degrading the xylan components of the plant cell wall and being assimilated by the pathogen. The nonhost plant *N. benthamiana* recognizes CfXyn11A in the apoplast through the plant membrane receptor complex, activating defense signal cascades to achieve NHR, whereas the host pear fails to recognize CfXyn11A. Simultaneously, the pear secretes PbXIP1 to inhibit the activity of CfXyn11A to enhance immunity, although it cannot function against other types of xylanases

In conclusion, this study provides a comprehensive characterization of apoplastic proteins and their expression levels in the *C. fructicola*–pear interaction, proposing a working model for CfXyn11A and PbXIP1 (Fig. 9). Upon detecting xylan components in the cell wall, *C. fructicola* secretes xylanases, including CfXyn11A, to degrade the cell wall and hydrolyze xylan for assimilation. The nonhost *N. benthamiana* recognizes CfXyn11A in the apoplast by a plant membrane receptor complex, activating defense signal cascades to achieve NHR, while the host pear fails to do so. Concurrently, pear secretes PbXIP1, which exhibits specific binding ability to inhibit CfXyn11A activity, thereby enhancing immunity.

## Materials and methods

### Plants, fungal strains, and treatments

The ‘Cuiguan’ pear tree specimens were sourced from the Jiangpu experimental orchard at Nanjing Agricultural University. Mature leaves were harvested from current-year shoots 20 days post-emergence between late May and early June. The selection criteria for leaves included uniform size, absence of disease or pest damage, and no prior pesticide exposure, ensuring their suitability as experimental material. The collected leaves underwent sterilization using a 0.1% sodium hypochlorite solution for 10 min. Following this, the leaves were thoroughly rinsed with distilled water 3–4 times to eliminate any residual sodium hypochlorite. These processed leaves were subsequently used for apoplastic fluid extraction and pathogenicity assays.

The VIGS and oxidative burst experiments utilized 35-day-old pear seedlings derived from ‘Cuiguan’ seeds. These seedlings were cultivated in a controlled greenhouse environment with a 16-h light and 8-h dark photoperiod, maintaining 75% relative humidity at a constant temperature of 25 °C.

The *C. fructicola* fungal strain NC40 used in this study was routinely cultured on PDA at 28 °C, as previously described (Li et al. 2022). To obtain fresh conidia, a 5-mm-diameter mycelial plug was placed in a 100-mL flask containing 50 mL of sterilized potato dextrose broth. The flasks were incubated at 28 °C with agitation at 180 rpm for 4 days. The concentration of the conidial suspension was quantified using a hemocytometer. Conidia were harvested, suspended in sterilized water, and diluted to a concentration of 1 × 10^4^ conidia/mL.

Leaves collected from the field for apoplastic fluid extraction were subjected to soaking inoculation with a conidial suspension. Fresh leaves were completely submerged in the conidial suspension for 30 min with gentle agitation, while the control group underwent mock-inoculation with pure water. The inoculated leaves were then incubated at 25 °C with 80% relative humidity. Five leaf samples were harvested at 12 hpi for RNA-seq analysis. These samples were immediately flash-frozen in liquid nitrogen and stored at − 80 °C. Samples collected for apoplastic fluid extraction were used in experiments without delay. This experiment was performed in triplicate.

### Apoplastic fluid extraction and LC–MS/MS

Apoplastic fluid extraction from infected leaves at 12 hpi followed a precise protocol. Initially, leaves were gently cleansed with distilled water to eliminate surface contaminants. The cleaned leaves were then immersed in 40 mL of ice-cold phosphate-buffered saline (PBS) buffer. A vacuum filtration system was used to apply − 80 hPa for 2-min intervals, alternating with slow returns to atmospheric pressure. This cycle was repeated five times to ensure buffer infiltration into the leaf intercellular spaces. Subsequently, the leaves were carefully rolled into a 50 mL syringe (without plunger) and placed in a 50-mL centrifuge tube, taking care to avoid leaf damage. Centrifugation was performed at 1500 × *g* for 45 min at 4 °C, with gradual acceleration and deceleration. The resulting apoplastic fluid was collected from the tube’s bottom and further purified through a 0.25 μm membrane column (Merck Millipore, St. Louis, MO, USA). A 10 kDa ultrafiltration tube (Merck Millipore) was used for higher concentrations.

Apoplastic protein identification was performed using LC–MS/MS by Shenzhen Huada Gene Technology Co., Ltd. (Shenzhen, China), as previously discussed (Han et al. 2024a). Data analysis was performed using Mascot v.2.3.02 (Matrix Science Inc., Boston, MA, USA), with searches conducted against the pear (*Pyrus bretschneideri*) and *C. fructicola* proteomes (Wang et al. 2023).

### RNA-seq and qRT-PCR

RNA extraction and sequencing were performed by Novogene Corporation (Nanjing, China). Total RNA was isolated using the fungi RNA Isolation Kit (Macrogen Inc., Seoul, South Korea). RNA purity was evaluated using the NanoPhotometer spectrophotometer (Implen Inc., Westlake Village, CA, USA), while concentration was determined using the Qubit RNA Assay Kit with the Qubit 2.0 Fluorometer (Life Technologies, Carlsbad, CA, USA). RNA integrity was assessed using the RNA Nano 6000 Assay Kit on the Bioanalyzer 2100 system (Agilent Technologies, Santa Clara, CA, USA). For sample preparation, 3 µg of RNA per sample was used. Sequencing libraries were generated using the NEBNext Ultra RNA Library Prep Kit for Illumina (NEB, Ipswich, MA, USA). These libraries were subsequently sequenced on an Illumina HiSeq platform, yielding 125 bp/150 bp paired-end reads.

Initial processing of raw data in FASTQ format was performed using in-house Perl scripts to generate clean reads. This process involved the removal of reads containing adapters, poly-N sequences, and low-quality reads. Quality metrics, including Q30, Q20, and GC content, were calculated for each sample. Subsequent analyses used the clean data. HISAT2 (Kim et al. 2015) was used to align the clean reads to the *C. fructicola* genome. FeatureCounts (Liao et al. 2014) was used to obtain read counts for each sample. TBtools (Chen et al. 2020) was then used to normalize read counts to tags per million.

qRT-PCR was performed using an Applied Biosystems StepOnePlus Real-Time PCR System with 2 × RealStar Green Power Mixture (GenStar, Beijing, China) in accordance with the manufacturer’s protocol. The comparative threshold cycle method, implemented with StepOne v.2.02, was used to calculate relative gene expression levels. Gene expression was standardized to the conidia data, using the β-tubulin gene as an internal reference. Each sample was analyzed with three technical replicates, and the complete experimental process, from leaf sample preparation to qRT-PCR analysis, was performed in triplicate.

### CfXyn11A and PbXIP1 secretion assay

The predicted N-terminal SP sequences of CfXyn11A and PbXIP1 were fused in-frame with the invertase gene in the pSUC2 vector. This vector contained the sucrose invertase gene SUC2 lacking the initiation ATG codon, and it was subsequently transformed into the yeast strain YTK12. EcoRI and XhoI restriction enzymes were used to insert the SP sequences into the pSUC2 vector. The transformed strains were cultured on yeast peptone dextrose agar (YPDA), Corn Meal Dextrose with Wickerham's vitamins (CMD-W), and selective YPRAA plates. YTK12 strains containing either the empty pSUC2 vector or pSUC2-Avr1bSP served as negative and positive controls, respectively. Enzyme activity was assessed by the reduction of 2,3,5-triphenyl tetrazolium chloride (TTC) to produce the red product TPF.

### Heterologous expression and purification of proteins

Linearized pPICZα plasmids containing CfXyn11A or its mutant were introduced into the *P. pastoris* X-33 strain by electroporation at 1.5 kV. Yeast cultures were cultivated on yeast extract-peptone-dextrose medium. For growth and protein induction, buffered glycerol-complex medium and buffered methanol-complex medium (EasySelect Pichia Expression Kit; Invitrogen, Carlsbad, CA, USA) at pH 6.5 were used, respectively. Protein expression was induced following the manufacturer’s protocol. The recombinant protein was isolated from the culture supernatant using Ni–NTA Superflow resin (Qiagen, Valencia, CA, USA) by affinity chromatography.

### Detection of the oxidative burst in plant leaves

Leaf disks (diameter 0.5 cm) extracted from 5-week-old *N. benthamiana* and 4-week-old pear plants were suspended in 200 µL of sterile water in a 96-well plate overnight. Subsequently, the water was replaced with 200 µL of reaction buffer containing luminol–peroxidase (35.4 mg/mL luminol, 10 mg/mL peroxidase, 1 mM CfXyn11A). Luminescence was quantified using the GLOMAX96 microplate luminometer (Promega, Madison, WI, USA). Measurement cycles (30 s each) were conducted for up to 50 min.

### Bioinformatics analysis

The genome and proteome sequences of 32 microorganisms and 4 plants were obtained from the National Center for Biotechnology Information (https://www.ncbi.nlm.nih.gov/) and Joint Genome Institute (https://mycocosm.jgi.doe.gov/). Hidden Markov model (HMM) profiles for the GH11 family (PF00457) were downloaded from Pfam (http://pfam.xfam.org). Subsequently, an HMM search was conducted on these proteome data using HMMER3. To eliminate redundant and incomplete sequences, overlapping genes were excluded, and the Conserved Domain Database (https://www.ncbi.nlm.nih.gov/cdd) was used to ensure the completeness of the conserved domains. Multiple sequence alignment was performed with MUSCLE, and the phylogenetic tree was generated using MEGA7.

### Enzyme activity assay

The xylanase activity was quantified using the DNS method. The standard reaction mixture comprised 10 μL of purified protein and 90 μL of 100 mM McIlvaine buffer (pH 5.5) containing 1% (w/v) beechwood xylan, incubated at 50 °C for 10 min. The reaction was halted by the addition of 150 μL of DNS solution. The mixture was subsequently boiled for precisely 5 min, cooled to room temperature, and its absorbance measured at 540 nm. A control reaction was performed by adding the enzyme sample after the DNS solution. All assays were performed in triplicate. One unit of xylanase activity was defined as the amount of enzyme necessary to liberate 1 μmol of reducing sugar per min from the substrate equivalent to xylose under the specified assay conditions (pH 5.5, 50 °C, 10 min).

The reducing sugar content of the apoplastic fluid of *N. benthamiana* transiently expressing CfXyn11A, CfXyn11A^E119D^, and CfXyn11A^E211D^ was measured using a previously established method (Ma et al. 2017). Apoplastic fluid was extracted from agroinfiltrated *N. benthamiana* leaves 36 h post-infiltration and sterilized by filtration. Subsequently, 10 μL of the filtered apoplastic fluid was transferred to wells in a second plate, followed by the addition of 100 μL DNS solution and 90 μL distilled water. To prevent evaporation, the plate was sealed with a microplate sealing film and incubated in a water bath at 100 °C for 5 min. After cooling, absorbance was measured at 540 nm using a SpectraMax® M5 microplate reader, with a blank control for reference. Xylose was used as a standard for calibration.

### CfXyn11A gene knockout and complementation

A split-marker approach was used to generate the CfXyn11A replacement cassette (Fig. S4). The upstream and downstream flanking sequences of CfXyn11A were amplified. The resulting PCR products were then fused with hygromycin phosphotransferase fragments using double-jointed PCR. These joined upstream and downstream products were subsequently transformed into *C. fructicola* protoplasts. Hygromycin (MDBio, Qingdao, China) was added to TB3 agar plates at a final concentration of 300 µg/mL for transformant selection. Transformants resistant to hygromycin were screened by PCR using four sets of primers (Fig. S4, Table S6). To complement the CfXyn11A mutant strain, the recombinant plasmid pFL2-CfXyn11A was introduced into the protoplasts of the CfXyn11A mutant strain. For the selection of transformants, G418 (Sigma-Aldrich, St. Louis, MO, USA) was added to TB3 agar plates at a final concentration of 400 µg/mL. Complemented strains were identified from G418-resistant transformants using PCR.

### Mycelial growth assay

Czapek agar plate medium served as the foundation, with various carbon sources incorporated at 2% (w/v), including glucose, sucrose, xylose, and xylan. The Czapek medium consisted of NaNO_3_ (3.0 g/L), KH_2_PO_4_ (1.0 g/L), MgSO_4_·7H_2_O (0.5 g/L), KCl (0.5 g/L), FeSO_4_·7H_2_O (0.01 g/L), and agar (15.0 g/L). *C. fructicola* WT strain and mutants were cultured at 28 °C for five days.

### IP–MS identified CfXyn11A host interactors

The coding sequences of CfXyn11A were inserted into pCN-GFP vectors, and both CfXyn11A-GFP and pCN-GFP constructs were introduced into *Agrobacterium* strain GV3101. *Agrobacterium* cultures containing each construct were resuspended in infiltration buffer to a final optical density (OD600) of 0.5 and co-infiltrated into *N. benthamiana* leaves. Two days post-infiltration, total proteins were extracted from the *N. benthamiana* leaves and incubated with ChromoTek GFP-Trap® Agarose (Proteintech, Chicago, IL, USA). The eluted proteins were separated on agarose gels and analyzed by LC–MS/MS. The resulting *N. benthamiana* protein sequences were aligned using BLASTp to identify pear apoplastic proteins, facilitating the screening of potential CfXyn11A interacting proteins.

### Protein interaction assays

For the LCI assays, the *CfXyn10A* and *CfXyn11A* genes were individually inserted into pCAMBIA1300-nLUC, while the pear PbXIP1 gene was inserted into pCAMBIA1300-cLUC. Six-week-old *N. benthamiana* plants were used for infiltration. *N. benthamiana* leaves were incubated with 1 mM luciferin substrate, and LUC images were captured 48 h post-infiltration using a Tanon-5200 Multi Chemiluminescent Imaging System (Tanon Science & Technology Co., Ltd., Shanghai, China).

In the GST pull-down assay, recombinant PbXIP1-GST protein was isolated from the Rosetta (DE3) *Escherichia coli* strain following isopropyl-beta-D-thiogalactopyranoside induction. The purified protein underwent incubation with 100 µL of glutathione-agarose beads (Yeasen Biotechnology Co., Ltd., Shanghai, China) at 4 °C for 4 h with agitation. After centrifugation at 6000 rpm for 5 min at 4 °C, the beads were collected and washed thrice with PBS. Subsequently, the beads were incubated with recombinant CfXyn11A-His protein at 4 °C for 2 h with agitation, followed by three washes with PBS. Finally, the beads were heated for 5 min at 100 °C in 40 µL of sodium dodecyl sulfate sample loading buffer, and the proteins were analyzed by immunoblotting with anti-His and anti-GST antibodies (Thermo Fisher Scientific, Waltham, MA, USA).

For the co-IP assay in *N. benthamiana*, the coding sequences of CfXyn11A and PbXIP1 were inserted into the vectors pCN-HA and pCN-Flag, respectively. The resulting CfXyn11A-HA and PbXIP1-Flag constructs were introduced into *Agrobacterium* strain GV3101. *Agrobacterium* cultures containing each construct were resuspended in infiltration buffer to a final OD600 of 0.5 and co-infiltrated into *N. benthamiana* leaves. Total proteins were extracted from the infiltrated *N. benthamiana* leaves 48 h post-infiltration and incubated with ChromoTek Flag-Trap® Agarose (Proteintech). Proteins eluted from the agarose beads were subsequently analyzed by immunoblotting with anti-HA (Thermo Fisher Scientific) or anti-Flag antibodies (Thermo Fisher Scientific).

### Construction of transgenic pear calli

*Agrobacterium*-mediated transformation was used to transform pear calli, following established protocols (Lin et al. 2023; Sun et al. 2024). The resulting kanamycin-resistant calli were subjected to subculture at three-week intervals under a selective pressure of 50 mg/L kanamycin. Confirmation of positive transgenic lines was achieved by DNA analysis, RNA analysis, and Western blotting techniques.

### VIGS assays in pear

VIGS was performed following established methods (Han et al. 2024a; Wang et al. 2024). A 384-bp PbXIP1 open reading frame was inserted into the EcoRI and BamHI sites of the TRV2 vector to create the PbXIP1-VIGS construct. The vectors pTRV1, pTRV2, and PbXIP1-pTRV2 were introduced into *A. tumefaciens* strain GV3101 by heat-shock transformation. Bacterial suspensions (OD600 = 1.0) containing pTRV1 were combined with either PbXIP1-pTRV2 or pTRV2 in a 1:1 volume ratio in 2-(morpholino)ethanesulfonic acid (MES) buffer (10 mmol/L MgCl_2_, 200 mmol/L acetosyringone, and 10 mmol/L MES, pH 5.6) and incubated with gentle agitation in darkness for 4 h at room temperature. The bacterial mixtures were subsequently injected into seedling leaves, which were then rinsed with water, planted in pots containing soil, and transferred to a controlled growth chamber. After two weeks, uninfected leaves from each plant were harvested for qRT-PCR analysis, and the VIGS plants exhibited comparable levels of PbXIP1 expression.

### *H*_*2*_*O*_*2*_* and O*_*2*_^*−*^* detection*

H_2_O_2_ and O_2_^−^ concentrations were measured using the Comin detection kit (Comin, Suzhou, China).

## Supplementary Information

Additional file 1: Fig. S1 The vacuum infiltration method for extracting apoplastic fluid from pear. A Leaves before and after infiltration. B Leaf weight alteration after vacuum infiltration. Values represent means (±SEM) (*n* = 15). Asterisks denote significant differences based on the Wilcoxon test (*P < 0.05). C The alteration in leaf weight after centrifugation and the weight of the extracted apoplastic fluid. Values represent means (±SEM) (*n* = 6). Asterisks denote significant differences based on the Wilcoxon test (ns, no significance).

Additional file 2: Fig. S2 Proteins identified in the apoplastic fluid lacking signal peptides were categorized based on Pfam annotation into glycosidases (blue), proteases (orange), lipases (green), oxidoreductases (yellow), and other proteins (gray). A *C. fructicola*. B Pear.

Additional file 3: Fig. S3 Relative expression of CfXyn11A in *C. fructicola* infected pear leaves.

Additional file 4: Fig. S4 Generation of CfXyn11A gene deletion mutants. A Schematic representation of gene deletion using the split-marker approach. B Primers used for gene replacement and screening. C Detection of deletion mutants using four primer pairs.

Additional file 5: death response in nine plant species triggered by 5 μM CfXyn11A or buffer. B ROS production in leaves of four distinct pear varieties treated with 200 nM flg22. Empty vector (EV) served as a negative control. Values represent means (±SEM) (*n*= 6). C ROS production in leaves of four distinct pear varieties treated with 1 μM CfXyn11A. EV served as a negative control. Values represent means (±SEM) (*n*= 6).

Additional file 6: Fig. S6 Disease lesions caused by *Phytophthora capsici* in *N. benthamiana*. A, C The leaves were pretreated 12 h before pathogen inoculation by infiltrating 50 nM CfXyn11Arec (A) or *C. fructicola conidia* (C). B, D Disease lesions were quantified at 36 h post-inoculation. Values represent means (±SEM) (*n*= 3).

Additional file 7: Fig. S7 Phenotypic response of *N. benthamiana* leaves after inoculation with *C. fructicola* wild-type strain or mutants (A) KO-8 and (B) KO-12.

Additional file 8: Fig. S8 Influence of pH (A) and temperature (B) on xylanase activity of purified CfXyn11A. Values represent means (±SEM) (*n*= 3).

Additional file 9: Fig. S9 Functional validation of PbXIP1 signal peptide. The strains were cultivated on YPDA, CMD-W, or YPRAA medium for 48 h. Invertase enzyme activity was evaluated by the conversion of TTC to insoluble TPF.

Additional file 10: Fig. S10 The capacity of CfXyn11A and PbXIP1 to elicit cell death in *N. benthamiana* after co-infiltration. PbXIP1 did not inhibit the cell death-inducing capacity of CfXyn11A in *N. benthamiana*.

Additional file 11: Fig. S11 Region G4 (positions 424–432) plays a crucial role in determining PbXIP1 association with CfXyn11A in plants. A The structural model of the PbXIP1–CfXyn11A complex based on AlphaFold and GRAMM. Color codes: cyan (PbXIP1), gray (CfXyn11A), and blue (predicted contact regions on PbXIP1). Four predicted contact regions on PbXIP1 (designated G1–G4) are listed. B Co-immunoprecipitation (Co-IP) assay examining the *in vivo* interaction between CfXyn11A and WT or five mutant PbXIP1 proteins. HA-tagged CfXyn11A was co-expressed with Flag-tagged WT or mutant PbXIP1 proteins in *N. benthamiana* leaves. IP was performed using anti-Flag affinity gel, followed by Western blot analysis with HA or Flag antibodies. Mutants G1–G4 contain alanine substitution mutations at the positions listed in (A). C Co-IP assay investigating the *in vivo* interaction between WT PbXIP1 and CfXyn11A enzyme activity proteins. HA-tagged WT or mutant CfXyn11A was co-expressed with Flag-tagged PbXIP1 proteins in *N. benthamiana* leaves. IP was performed using anti-Flag affinity gel, followed by Western blot analysis with HA or Flag antibodies.

Additional file 12: Table S1 *C. fructicola* proteins identified by LC–MS/MS analysis.

Additional file 13: Table S2 Pear proteins identified by LC–MS/MS analysis.

Additional file 14: Table S3 RNA-seq read counts and mapping rates of samples.

Additional file 15: Table S4 GO enrichment analysis of *C. fructicola* apoplastic proteins.

Additional file 16: Table S5 Apoplastic proteins predicted by mass spectrometry analysis, obtained from total proteins immunoprecipitated after transient expression in *N. benthamiana* leaves, along with their orthologs identified in pear apoplastic fluid.

Additional file 17: Table S6 List of primers.

## Data Availability

The datasets generated in this study have been deposited in the China National GeneBank (CNGB) Sequence Archive and are accessible at https://db.cngb.org/cnsa/. The accession number for these datasets is CNP0005915.

## Abbreviations

BMGY: Buffered glycerol-complex medium
BMMY: Buffered methanol-complex medium
Ct: Comparative threshold cycle
CWDEs: Cell wall-degrading enzymes
DAMPs: Damage-associated molecular patterns
DNS: 3,5-Dinitrosalicylic acid
EV: Empty vector
GFP: Green fluorescent protein
GH: Glycosyl hydrolase
GO: Gene Ontology
GST: Glutathione S-transferase
Hph: Hygromycin phosphotransferase
Hpi: Hours post inoculation
IH: Intracellular hyphae
LC–MS/MS: Liquid Chromatography-Tandem Mass Spectrometry
LUC: Luciferase
LRR: Leucine-rich repeat
MAMPs: Microbial-associated molecular patterns
NHR: Nonhost resistance
PDA: Potato dextrose agar
PDB: Potato dextrose broth
PBS: Phosphate-buffered saline
PCD: Programmed Cell Death
PGIP: Polygalacturonase-inhibiting protein
PR: Pathogenesis-related protein
PRRs: Pattern recognition receptors
PTI: PAMP-triggered immunity
qRT-PCR: Quantitative Real Time PCR
ROS: Reactive oxygen species
SDS: Sodium dodecyl sulfate
SH: Secondary necrotrophic hyphae
SP: Signal peptide
TAXI: *Triticum aestivum* Xylanase-inhibitor
TPF: 1,3,5-Triphenylformazan
TPM: Tags per million
TRV: Virus-based vector
TTC: 2,3,5-Triphenyl tetrazolium chloride
VIGS: Virus-induced gene silencing
WT: Wild-type
YEPD: Yeast extract-peptone-dextrose
