# Supplementary figures and images for: Apoplastic proteomic reveals Colletotrichum fructicola effector CfXyn11A recognized by tobacco and suppressed by pear in the apoplast
Source: Mol Hortic. 2025 Jul 7;5:42. doi: 10.1186/s43897-025-00161-3 (PMC12232838; doi:10.1186/s43897-025-00161-3)

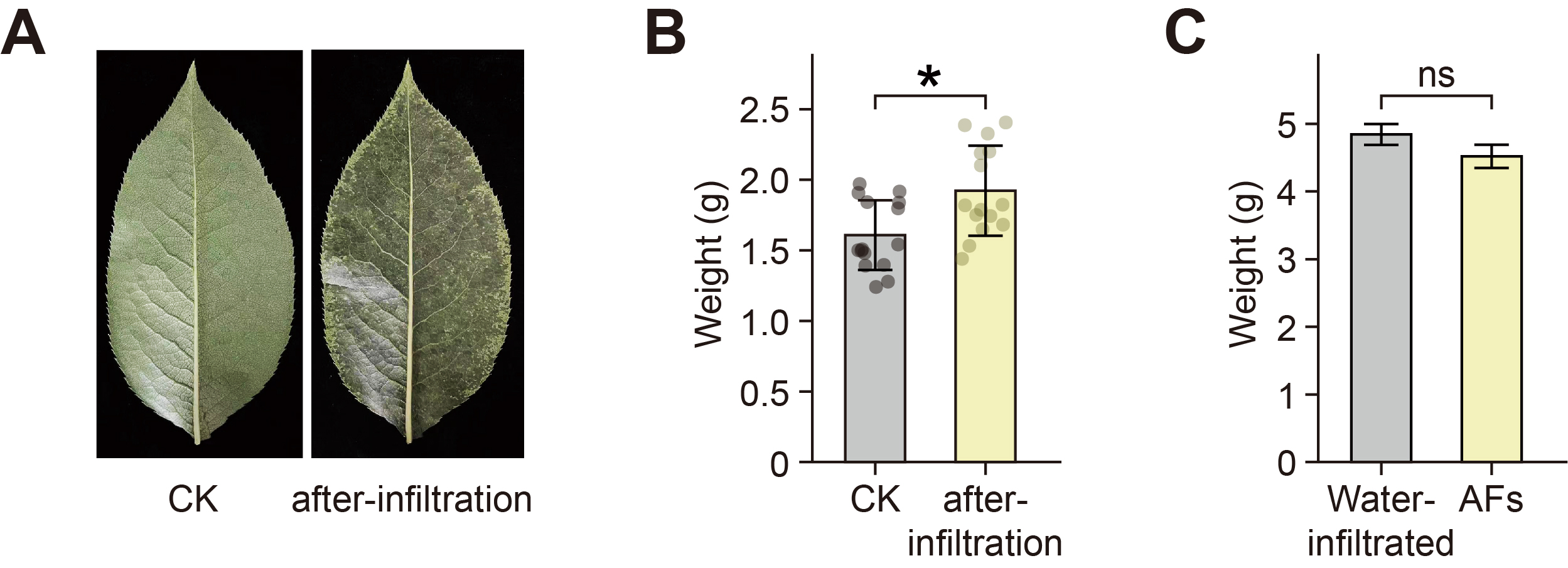

Supplement: Supplementary file 1 — Additional file 1: Fig. S1 The vacuum infiltration method for extracting apoplastic fluid from pear. A Leaves before and after infiltration. B Leaf weight alteration after vacuum infiltration. Values represent means (±SEM) (n = 15). Asterisks denote significant differences based on the Wilcoxon test (*P < 0.05). C The alteration in leaf weight after centrifugation and the weight of the extracted apoplastic fluid. Values represent means (±SEM) (n = 6). Asterisks denote significant differences based on the Wilcoxon test (ns, no significance). [file 43897_2025_161_MOESM1_ESM.jpg]

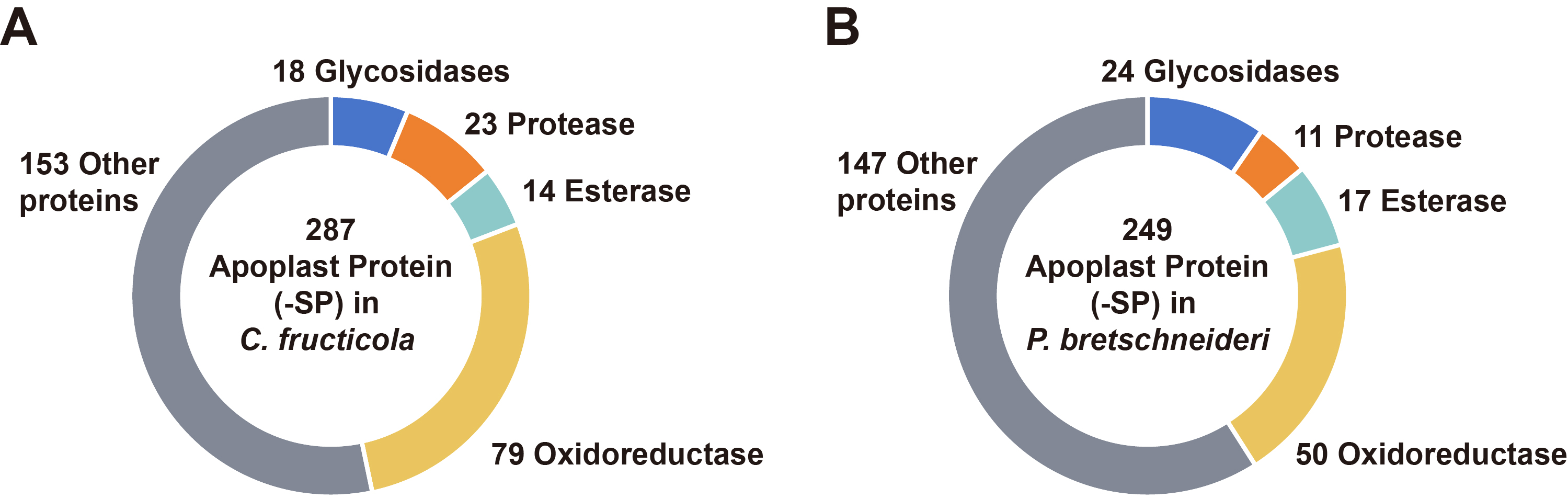

Supplement: Supplementary file 2 — Additional file 2: Fig. S2 Proteins identified in the apoplastic fluid lacking signal peptides were categorized based on Pfam annotation into glycosidases (blue), proteases (orange), lipases (green), oxidoreductases (yellow), and other proteins (gray). A C. fructicola. B Pear. [file 43897_2025_161_MOESM2_ESM.jpg]

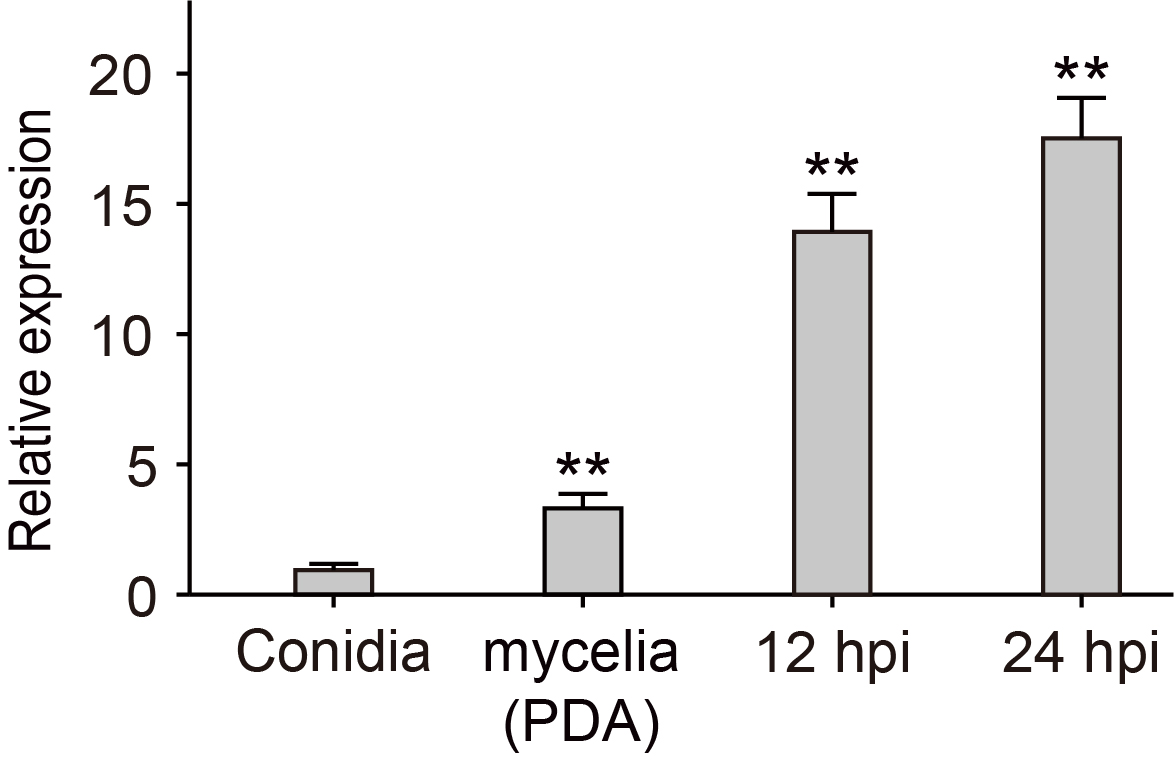

Supplement: Supplementary file 3 — Additional file 3: Fig. S3 Relative expression of CfXyn11A in C. fructicola infected pear leaves. [file 43897_2025_161_MOESM3_ESM.jpg]

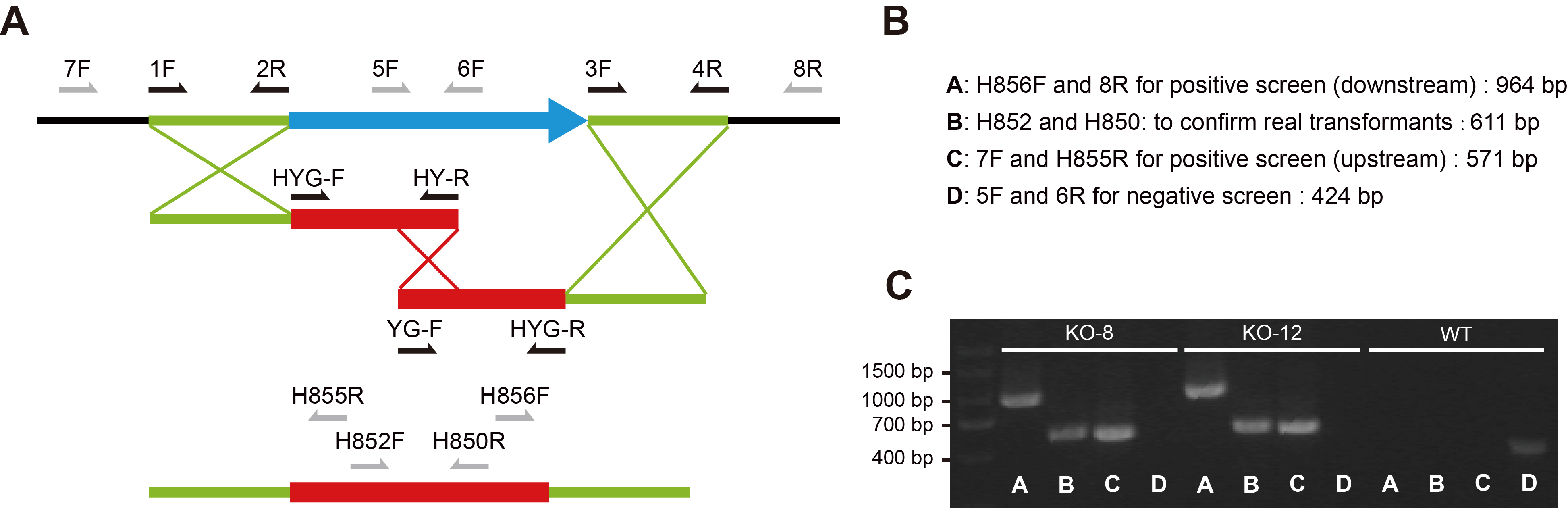

Supplement: Supplementary file 4 — Additional file 4: Fig. S4 Generation of CfXyn11A gene deletion mutants. A Schematic representation of gene deletion using the split-marker approach. B Primers used for gene replacement and screening. C Detection of deletion mutants using four primer pairs. [file 43897_2025_161_MOESM4_ESM.jpg]

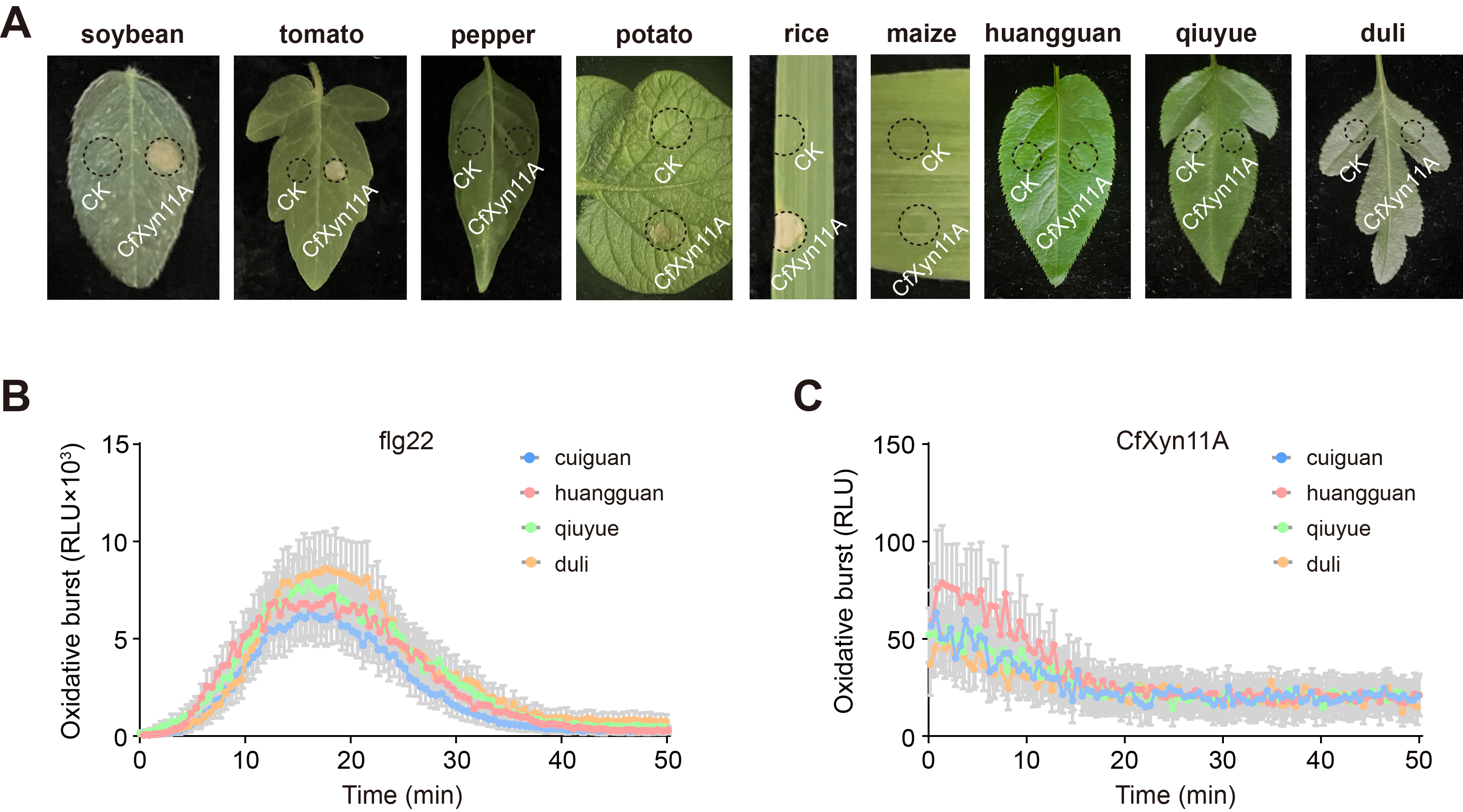

Supplement: Supplementary file 5 — Additional file 5: death response in nine plant species triggered by 5 μM CfXyn11A or buffer. B ROS production in leaves of four distinct pear varieties treated with 200 nM flg22. Empty vector (EV) served as a negative control. Values represent means (±SEM) (n= 6). C ROS production in leaves of four distinct pear varieties treated with 1 μM CfXyn11A. EV served as a negative control. Values represent means (±SEM) (n= 6). [file 43897_2025_161_MOESM5_ESM.jpg]

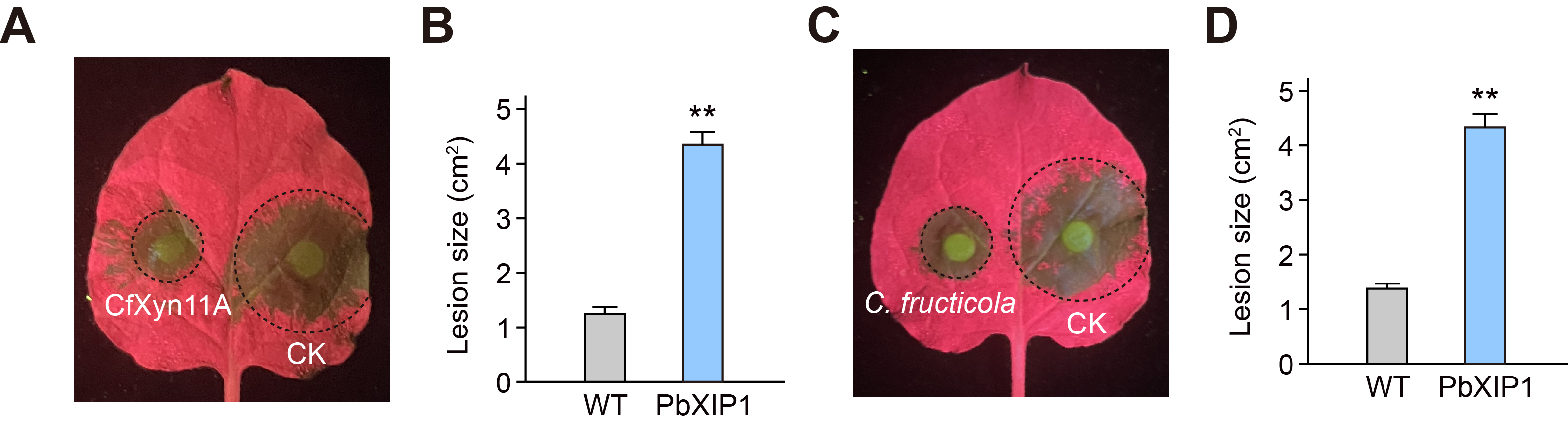

Supplement: Supplementary file 6 — Additional file 6: Fig. S6 Disease lesions caused by Phytophthora capsici in N. benthamiana. A, C The leaves were pretreated 12 h before pathogen inoculation by infiltrating 50 nM CfXyn11Arec (A) or C. fructicola conidia (C). B, D Disease lesions were quantified at 36 h post-inoculation. Values represent means (±SEM) (n= 3). [file 43897_2025_161_MOESM6_ESM.jpg]

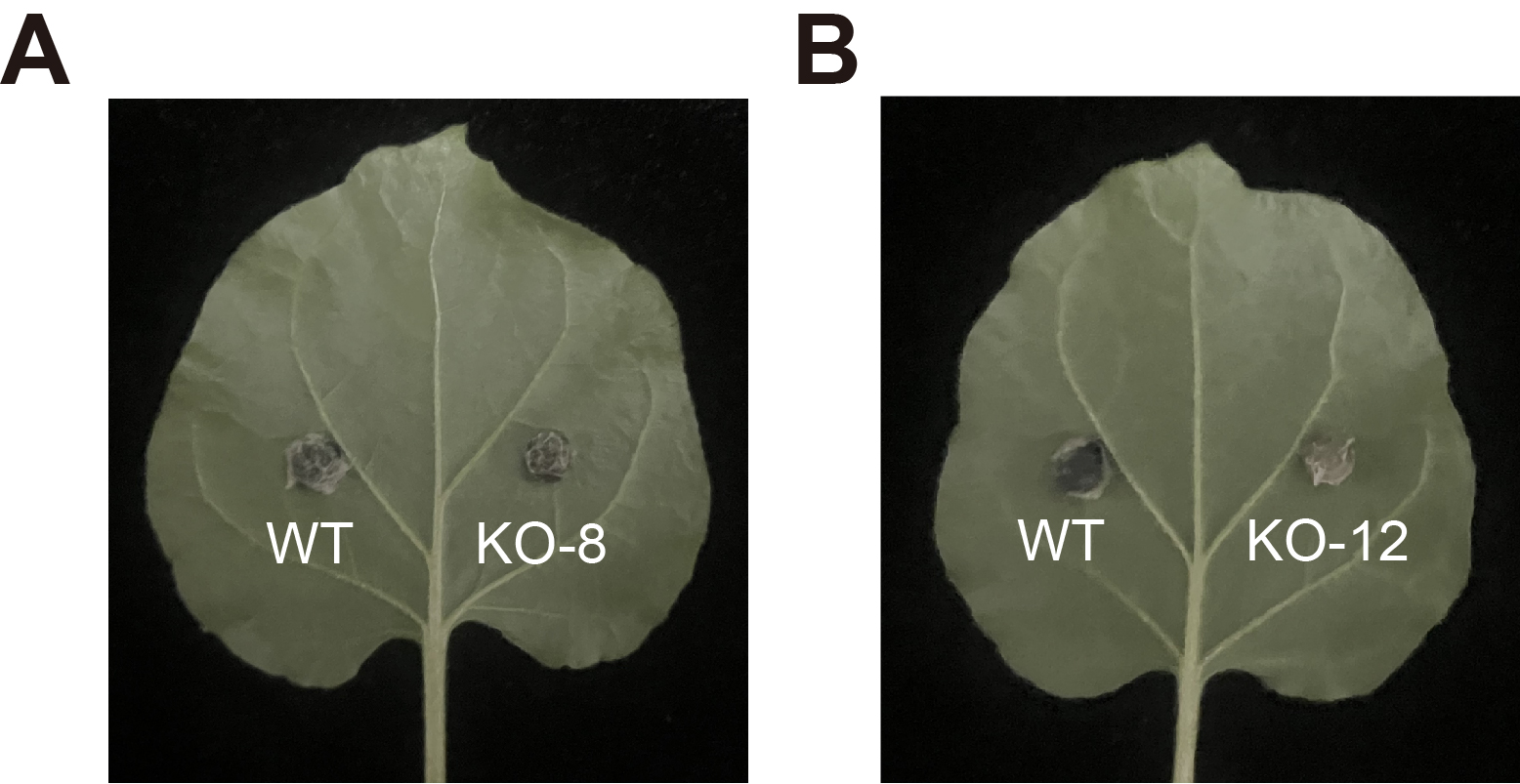

Supplement: Supplementary file 7 — Additional file 7: Fig. S7 Phenotypic response of N. benthamiana leaves after inoculation with C. fructicola wild-type strain or mutants (A) KO-8 and (B) KO-12. [file 43897_2025_161_MOESM7_ESM.jpg]

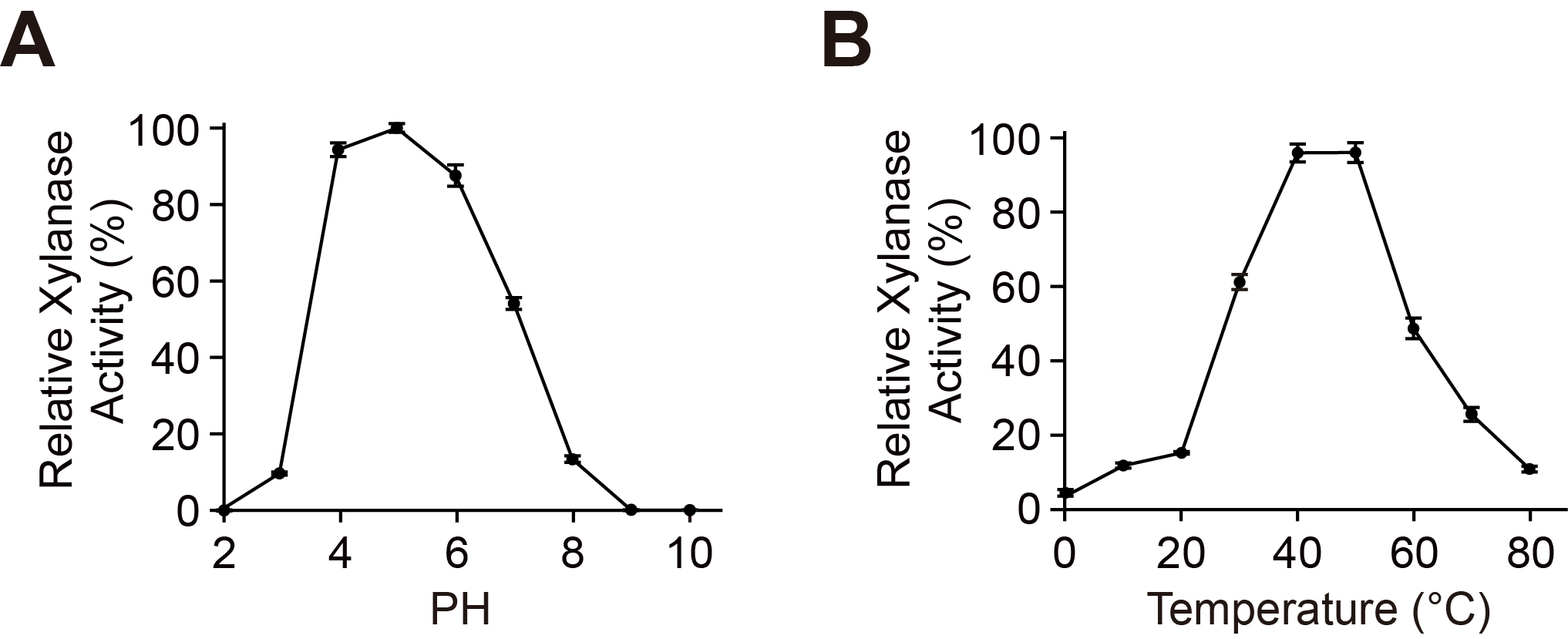

Supplement: Supplementary file 8 — Additional file 8: Fig. S8 Influence of pH (A) and temperature (B) on xylanase activity of purified CfXyn11A. Values represent means (±SEM) (n= 3). [file 43897_2025_161_MOESM8_ESM.jpg]

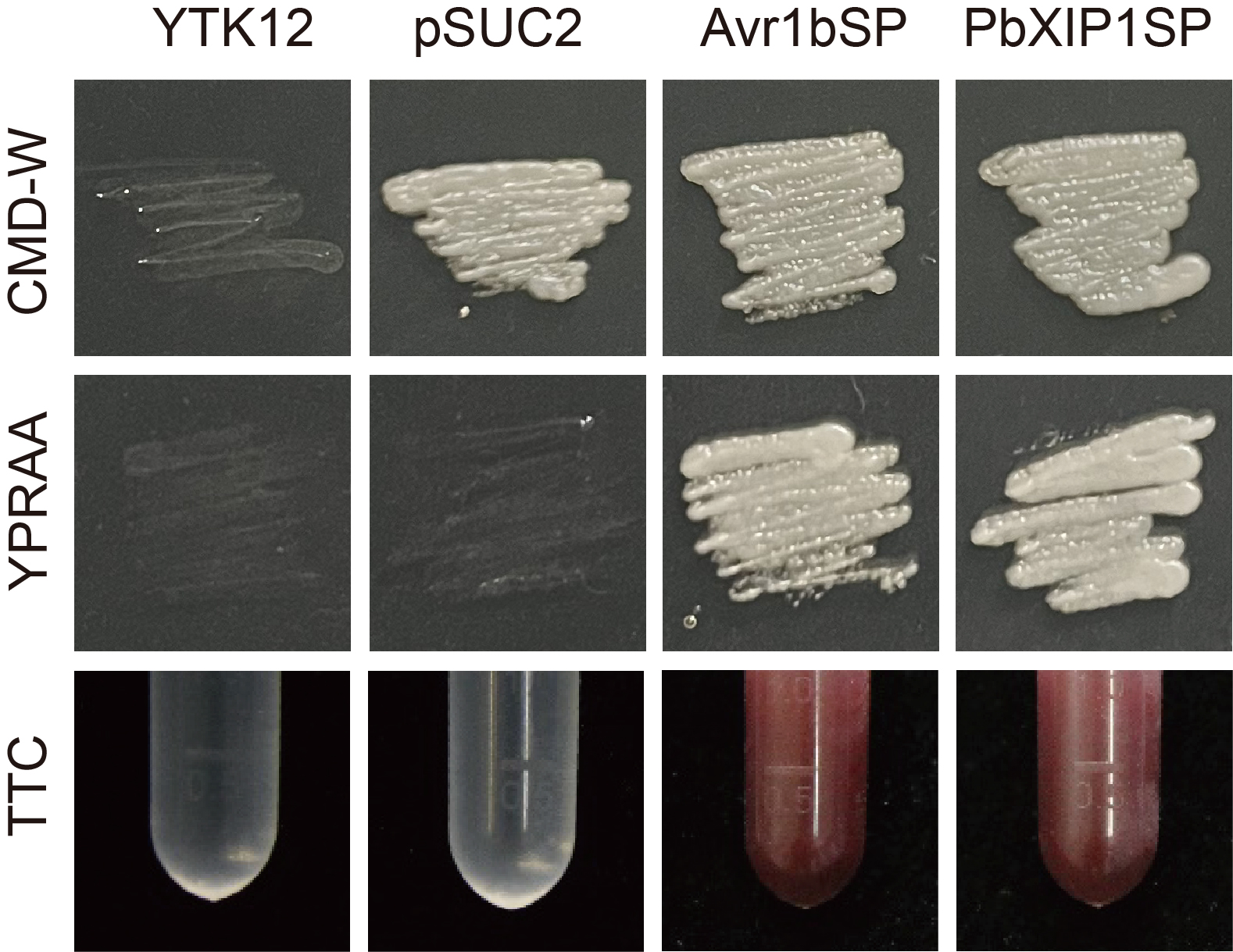

Supplement: Supplementary file 9 — Additional file 9: Fig. S9 Functional validation of PbXIP1 signal peptide. The strains were cultivated on YPDA, CMD-W, or YPRAA medium for 48 h. Invertase enzyme activity was evaluated by the conversion of TTC to insoluble TPF. [file 43897_2025_161_MOESM9_ESM.jpg]

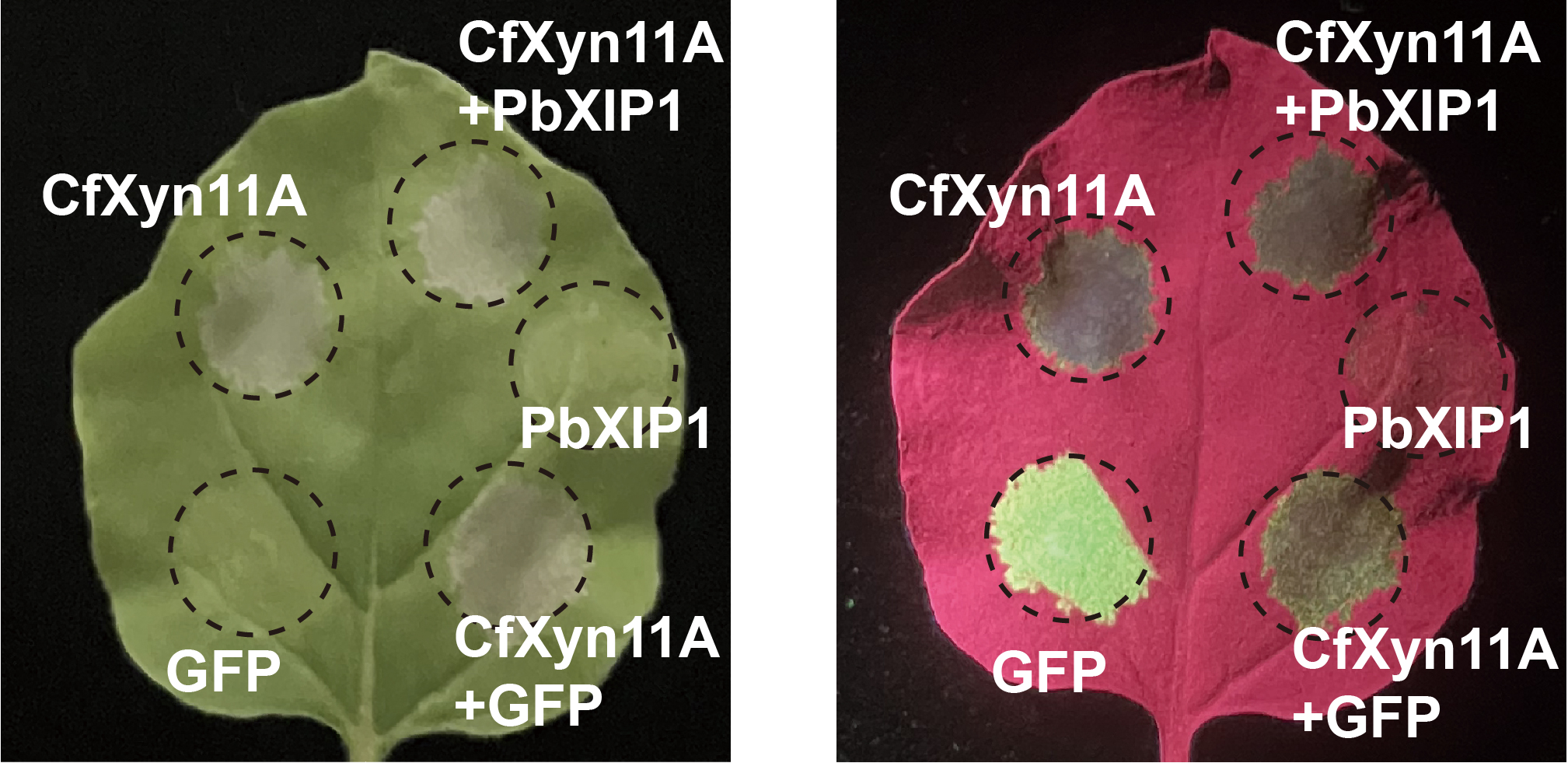

Supplement: Supplementary file 10 — Additional file 10: Fig. S10 The capacity of CfXyn11A and PbXIP1 to elicit cell death in N. benthamiana after co-infiltration. PbXIP1 did not inhibit the cell death-inducing capacity of CfXyn11A in N. benthamiana. [file 43897_2025_161_MOESM10_ESM.jpg]

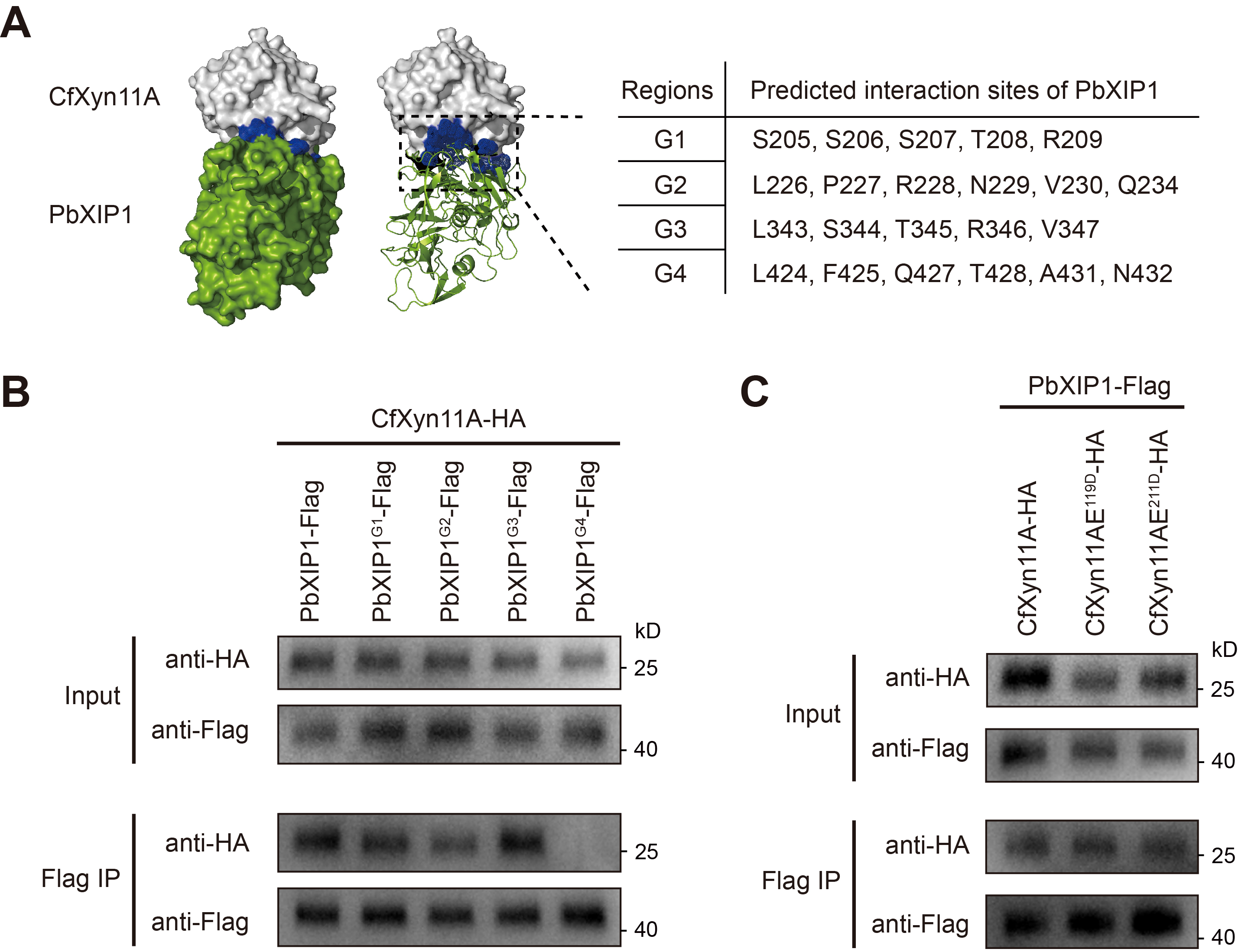

Supplement: Supplementary file 11 — Additional file 11: Fig. S11 Region G4 (positions 424–432) plays a crucial role in determining PbXIP1 association with CfXyn11A in plants. A The structural model of the PbXIP1–CfXyn11A complex based on AlphaFold and GRAMM. Color codes: cyan (PbXIP1), gray (CfXyn11A), and blue (predicted contact regions on PbXIP1). Four predicted contact regions on PbXIP1 (designated G1–G4) are listed. B Co-immunoprecipitation (Co-IP) assay examining the in vivo interaction between CfXyn11A and WT or five mutant PbXIP1 proteins. HA-tagged CfXyn11A was co-expressed with Flag-tagged WT or mutant PbXIP1 proteins in N. benthamiana leaves. IP was performed using anti-Flag affinity gel, followed by Western blot analysis with HA or Flag antibodies. Mutants G1–G4 contain alanine substitution mutations at the positions listed in (A). C Co-IP assay investigating the in vivo interaction between WT PbXIP1 and CfXyn11A enzyme activity proteins. HA-tagged WT or mutant CfXyn11A was co-expressed with Flag-tagged PbXIP1 proteins in N. benthamiana leaves. IP was performed using anti-Flag affinity gel, followed by Western blot analysis with HA or Flag antibodies. [file 43897_2025_161_MOESM11_ESM.jpg]
